# Cloning and Characterization of a Wheat Homologue of Apurinic/Apyrimidinic Endonuclease Ape1L

**DOI:** 10.1371/journal.pone.0092963

**Published:** 2014-03-25

**Authors:** Botagoz Joldybayeva, Paulina Prorok, Inga R. Grin, Dmitry O. Zharkov, Alexander A. Ishenko, Barbara Tudek, Amangeldy K. Bissenbaev, Murat Saparbaev

**Affiliations:** 1 Department of Molecular Biology and Genetics, Faculty of Biology, al-Farabi Kazakh National University, Almaty, Kazakhstan; 2 Groupe «Réparation de l'ADN», CNRS UMR8200, Université Paris-Sud, Institut Gustave Roussy, Villejuif, France; 3 Institute of Biochemistry and Biophysics, Polish Academy of Sciences, Warsaw, Poland; 4 SB RAS Institute of Chemical Biology and Fundamental Medicine, Novosibirsk, Russia; 5 Novosibirsk State University, Novosibirsk, Russia; 6 Institute of Genetics and Biotechnology, University of Warsaw, Warsaw, Poland; University of Pittsburgh, United States of America

## Abstract

**Background:**

Apurinic/apyrimidinic (AP) endonucleases are key DNA repair enzymes involved in the base excision repair (BER) pathway. In BER, an AP endonuclease cleaves DNA at AP sites and 3′-blocking moieties generated by DNA glycosylases and/or oxidative damage. A *Triticum aestivum* cDNA encoding for a putative homologue of ExoIII family AP endonucleases which includes *E. coli* Xth, human APE1 and *Arabidopsis thaliana* AtApe1L has been isolated and its protein product purified and characterized.

**Methodology/Principal Findings:**

We report that the putative wheat AP endonuclease, referred here as TaApe1L, contains AP endonuclease, 3′-repair phosphodiesterase, 3′-phosphatase and 3′→5′ exonuclease activities. Surprisingly, in contrast to bacterial and human AP endonucleases, addition of Mg^2+^ and Ca^2+^ (5–10 mM) to the reaction mixture inhibited TaApe1L whereas the presence of Mn^2+^, Co^2+^ and Fe^2+^ cations (0.1–1.0 mM) strongly stimulated all its DNA repair activities. Optimization of the reaction conditions revealed that the wheat enzyme requires low divalent cation concentration (0.1 mM), mildly acidic pH (6–7), low ionic strength (20 mM KCl) and has a temperature optimum at around 20°C. The steady-state kinetic parameters of enzymatic reactions indicate that TaApe1L removes 3′-blocking sugar-phosphate and 3′-phosphate groups with good efficiency (*k*_cat_/*K*_M_ = 630 and 485 μM^−1^·min^−1^, respectively) but possesses a very weak AP endonuclease activity as compared to the human homologue, APE1.

**Conclusions/Significance:**

Taken together, these data establish the DNA substrate specificity of the wheat AP endonuclease and suggest its possible role in the repair of DNA damage generated by endogenous and environmental factors.

## Introduction

Cellular DNA is a target for various endogenous and exogenous factors that alter its primary structure and can lead to mutations and/or cell death. Importantly, DNA damage can be induced by specialized cellular enzymes during various biological processes such as erasure of DNA cytosine methylation patterns and immunoglobulin genes maturation [1], [2]. To keep genome integrity and to assure faithful transfer of genetic information during cell division, living organisms develop several distinct DNA repair systems that remove and/or tolerate the DNA lesions. Until now, molecular characterization of the DNA repair mechanisms have been mainly focused on *E. coli*, yeast and mammalian cells [3], whereas little is known on the mechanisms that maintain genome stability in plants. Green plants are constantly exposed to ultraviolet and other environmental factors which extensively damage cellular DNA. In addition, plants generate reactive oxygen species (ROS) during respiration in mitochondria and during photosynthesis in chloroplasts. Oxidative damage to DNA caused by ROS is believed to be a major type of endogenous cellular damage [4]. Oxidative DNA lesions are substrates for two overlapping pathways: base excision repair (BER) and nucleotide incision repair (NIR) [5], [6]. In the classic BER pathway, a DNA glycosylase hydrolyses the *N*-glycosydic bond between the damaged base and the sugar, leaving either an apurinic/apyrimidinic (AP) site or a single-stranded DNA break or a 1-nt gap flanked with a 3′-blocking group and 5′-phosphate [7]. Alternatively, in the NIR pathway, an AP endonuclease makes an incision 5′ next to a damaged nucleotide and generates a single-strand break with a 3′-hydroxyl group and a 5′-dangling modified nucleotide [8].

Plant genome undergoes dramatic changes in its DNA cytosine methylation pattern during development and in response to environmental factors [9], [10]. The level of 5-methylcytosine (5 mC) residues in genome is dependent on both DNA methylation and demethylation processes. Genetic and biochemical evidences indicate that plants contain several bi-functional DNA glycosylases/AP lyases that specifically excise 5 mC when present in duplex DNA and initiate its replacement by regular cytosine in the BER pathway [11]. In *Arabidopsis thaliana* a family of 5mC-DNA glycosylases, ROS1, DME, DML2, DML3 are involved in the regulation of imprinting and gene silencing [11]. These enzymes contain, in addition to DNA glycosylase, an AP lyase activity that cleaves the AP site generated after 5 mC excision by β elimination and/or β/δ elimination reactions [12]. The final product of 5mC-DNA glycosylases action is either a nick flanked with a 5′-phosphate and a 3′-phospho α,β-unsaturated aldehyde (3′-PA), or a single-nucleotide gap flanked with a 5′-phosphate and a 3′-phosphate (3′-P) [12]. Thus, the plant 5mC-DNA glycosylase-initiated active DNA demethylation generates highly genotoxic DNA strand breaks containing non-hydroxyl 3′-groups, which cannot be used by DNA polymerases and DNA ligases and therefore should be removed before initiating DNA repair synthesis.

The genome of *Arabidopsis thaliana*, a widely used model plant organism, contains three genes encoding homologues of human major AP endonuclease 1 (APE1): *Arp*, *Ape1L* and *Ape2*. At present, only the Arp protein has been characterized whereas it is unknown whether Ape1L and Ape2 have any DNA repair activity. Arp is a hydrolytic class II AP endonuclease that cleaves duplex DNA 5′ next to AP site and generates a single-strand break flanked with a 3′-hydroxyl (3′-OH) and a 5′-deoxyribose-phosphate (5′-dRp) [13]. In addition, Arp contains a redox activity analogous to the redox activity of human APE1 that stimulates DNA binding by AP-1 and other transcription factors. Recently, it has been demonstrated that in the presence of Mg^2+^, Arp represents the major AP site cleavage activity in *Arabidopsis* cell-free extracts [14], [15]. Interestingly, *Arp* ^–/–^ mutants grow normally and do not display phenotypic defects under laboratory conditions, however, they are hypersensitive to the presence of 5-fluorouracil (5-fU) in the growth medium suggesting that Arp is essential when an increased number of AP sites is generated by the mono-functional uracil-DNA glycosylase (AtUNG) [15]. Importantly, in *Arabidopsis* cell-free extracts, in the absence of Mg^2+^, the AP sites generated by AtUNG are mainly processed by AP lyases yielding single-strand breaks with a mixture of 3′-P and 3′-PA termini [14]. Furthermore, in the absence of Arp, AP sites and 5 mC can be removed by combined action of ROS1, which cleaves AP sites to generate strand breaks containing either 3′-P or 3′-PA termini, and the Zinc finger DNA 3′-Phosphatase (ZDP) which removes 3′-P, but not 3′-PA to produce 3′-OH termini for DNA repair synthesis and ligation [16]. The 3′-PA termini generated by ROS1 after removal of 5 mC residues are processed by an unknown 3′-repair phosphodiesterase in an Arp- and ZDP-independent manner [16].

A genetic study conducted by Murphy and colleagues showed that *A. thaliana* plants deficient for any one of *Arp*, *Ape1L*, or *Ape2* genes exhibit no obvious phenotypic deviations from the wild type [17]. However, double knock-out mutations in *Ape1L* and *Ape2* genes are lethal resulting in a seed-abortion phenotype, whereas mutations in *Arp* in combination with either *Ape1L* or *Ape2* are not deleterious [17]. These observations suggest that Ape1L and Ape2 are required to repair DNA damage that occurs during seed development and/or for the 3′-end processing during active DNA demethylation initiated by 5mC-DNA glycosylases. To date no information is available on the biochemical properties and DNA substrate specificities of the plant Ape1L and Ape2 proteins, nor on the presence of 3′-repair phosphatase or phosphodiesterase activities in all three plant AP endonucleases.

*Triticum aestivum* (wheat) is a major renewable resource for human food, domestic animal feed, and industrial raw materials. The complexity of the crop's genome in terms of size (17 Gb for the bread wheat genome, i.e., five times the human genome and fourty times the rice genome), repeat content (>80%), and ploidy (hexaploid) has often been considered too challenging for efficient molecular studies [18]. At present scarce data are available regarding the characterization of wheat DNA glycosylases and AP endonucleases. So far only the uracil-DNA glycosylase from wheat germ has been characterized biochemically [19]. More recently, we have identified an AP endonuclease activity in extracts from wheat aleurone layer cells that cleaves DNA duplexes containing a synthetic AP site and alpha-anomer of 2′-deoxyadenosine (αdA) residue suggesting that the BER and NIR pathways are evolutionary conserved in wheat [20]. Based on the available DNA sequence data we have isolated and cloned *T. aestivum* cDNA gene encoding a putative AP endonuclease, referred here as TaApe1L, which is homologous to human major AP endonuclease 1 (APE1) and *A. thaliana* AtApe1L. DNA substrate specificity of the homogenous wheat TaApe1L protein and its biological roles will be discussed.

## Materials and Methods

### Chemicals, reagents and proteins

Cell culture media were purchased from Invitrogen (Life Technologies SAS, Saint Aubin, France). Restriction enzymes and T4 DNA ligase were from New England Biolabs France (Evry, France). The *E. coli* BL21(DE3) Rosetta cells were from Novagen-EMD4 Biosciences (Merck Chemicals, Nottingham, UK).

The activities of various DNA repair proteins were tested using their primary substrates immediately before use. The purified DNA proteins including *E. coli* formamidopyrimidine-DNA glycosylase (Fpg), endonuclease III (Nth), endonuclease IV (Nfo) and human catalytic domain uracil-DNA glycosylase (hUNG) and major human AP endonuclease 1 (APE1) were from the laboratory stock [6], [21]. The purified human POLβ was purchased from Trevigen (Gaithersburg, USA).

### Oligonucleotides

Oligodeoxyribonucleotides containing modified residues, and their complementary oligonucleotides were purchased from Eurogentec (Seraing, Belgium). They included 30-mers d(TGACTGCATAXGCATGTAGACGATGTGCAT) where X is either tetrahydrofuran (THF, an abasic site analogue), uracil (U), alpha-2′-deoxyadenosine (αdA) or 5,6-dihydrouracil (DHU) for kinetic studies, and 30-mer complementary oligonucleotides containing either dA, dG, dC or T opposite the lesion. A 34-mer d(AAATACATCGTCACCTGGGUCATGTTGCAGATCC) annealed to its complementary strand containing dG opposite to U was treated by uracil-DNA glycosylase and then by AP lyases or AP endonucleases to generate single-strand nicks containing 3′-blocking groups. These sequence contexts were previously used to study the nucleotide incision activities of bacterial and yeast AP endonucleases [22].

The following oligonucleotides were used to measure 3′→5′ exonuclease and 3′-repair phosphatase and phosphodiesterase activities: Exo20, d(GTGGCGCGGAGACTTAGAGA); Exo20^THF^, d(GTGGCGCGGAGACTTAGAGAX), where X is a 3′-terminal THF; Exo20^P^, d(GTGGCGCGGAGACTTAGAGAp), where p is 3′-terminal phosphate; 5P-Exo19, d(pATTTGGCGCGGGGAATTCC), where p is 5′-terminal phosphate; and complementary 40 mer Rex-G, d(GGAATTCCCCGCGCCAAATGTCTCTAAGTCTCCGCGCCAC). The nicked/gapped duplexes, Exo20•G, Exo20^THF^•G, Exo20^P^•G were comprised of 5P-Exo19 and Rec-G, and Exo20, or Exo20^P^, or Exo20^THF^, respectively. These sequence contexts were previously used to study the 3′-repair activities of bacterial, yeast and human AP endonucleases [23].

The oligonucleotides were 5′-labelled by phosphatase minus mutant of T4 polynucleotide kinase (New England Biolabs) in the presence of γ[^32^P]ATP (3,000 Ci/mmol) (PerkinElmer) as recommended by the manufacturers. The labelled oligonucleotides were annealed to their appropriate complementary oligonucleotides in a buffer containing 50 mM NaCl, 10 mM HEPES-KOH (pH 7.5) at 65°C for 3 min as previously described [8].

### RNA isolation and cDNA synthesis

The *T. aestivum* cDNA econding putative AP endonuclease (*taape1*) was identified by its homology to the human Ape1 (AAH02338) and Arabidopsis Ape1L genes (At3g48425). Using the cDNA sequence of putative *taape1* from Genbank accession AK333560, the forward primer, *taape1* Dir: d(CTGCACTCATATGAAGCGCTTCTTCCAGCC) and the reverse primer, *taape1* Rev: d(CCAGGATCCTTAGCCGGAGCTCTTCGATTC) were designed and used for RT-PCR. The underlined bases in primers are the restriction sites of *Nde*I and *Bam*HI endonucleases, respectively.

Total RNA was isolated from young leaves of *T. aestivum* variety Kazakhstanskaya 10 by trizol method. The yield and purity of RNA was determined spectrophotometrically and the quality of RNA was tested on 1% formaldehyde agarose gel. The first strand of cDNA was synthesized by reverse transcription using 5 μg of total RNA as a template under the following conditions: 200 U RevertAid M-MuLV reverse transcriptase (Thermo Scientific, Lithuania) with 0.5 μg of oligo-dT_18_ primer, 1 mM dNTPs in a final volume of 20 μl. An aliquot of the first cDNA strand was used as a template in the PCR reaction for the synthesis of second strand of cDNA and the subsequent amplification of double-stranded cDNA was performed with the designed gene-specific primers. The PCR was carried out as follows: predenaturation at 95°C, 2 min; 25 cycles of denaturation at 95°C, 30 s; annealing at 58°C, 45 s; extension at 72°C, 1.5 min and a final extension step at 72°C for 10 min. The amplified products were separated on a 1% agarose gel and the product of expected size was extracted from the gel using Silica Bead DNA Gel Extraction kit (Thermo Scientific, Lithuania). The fragment was cloned into the pBluescriptII SK(+) vector at *Nde*I and *BamH*I restriction sites using the Rapid DNA ligation kit (Thermo Scientific, Lithuania) and the ligation product was transformed into competent *E. coli* DH5α cells. Transformed colonies carrying the plasmid with an insert were screened by complementation of *lacZ* gene and the plasmid DNAs were isolated with GeneJET Plasmid Miniprep kit (Thermo Scientific, Lithuania). The presence of the insert in the isolated plasmids was confirmed by PCR with gene-specific primers, and its sequence was confirmed by sequencing in both directions with M13 forward and reverse primers.

### Expression and purification of His-tagged TaApe1L

The DNA fragment encoding TaApe1L was subcloned into the pET28c vector at the *Nde*I-*BamH*I sites resulting in the expression plasmid pET28c-TaApe1L which carries an N-terminal His-tag sequence. The wheat TaApe1L protein was expressed and purified from *E. coli* Rosetta(DE3) strain. Briefly, *E. coli* cells were electroporated with pET28c-TaApe1L, the resulting kanamycin-resistant transformants were grown to OD_600_  = 0.6 at 37°C, and protein overproduction was then induced by 0.1 mM isopropyl β-D-1-thiogalactopyranoside (IPTG) overnight at 30°C. Due to high-level expression in the Rosetta strain, it was possible to purify TaApe1L to homogeneity using only two chromatographic steps. All purification procedures were carried out at 4°C. Bacteria were harvested by centrifugation and cell pellets were lysed using a French press at 18,000 psi in a buffer containing 20 mM HEPES-KOH (pH 7.6), 50 mM KCl supplemented with Complete Protease Inhibitor Cocktail (Roche Diagnostics, Switzerland). The lysates were cleared by centrifugation at 40,000×*g* for 30 min at 4°C, the resulting supernatant was adjusted to 500 mM NaCl and 20 mM imidazole and loaded onto a HiTrap Chelating HP column (GE Healthcare) charged with Ni^2+^. The eluted fractions containing TaApe1L were pooled and loaded onto a 1-ml HiTrap-Heparin column (GE Healthcare). The bound proteins were eluted in a 50–600 mM KCl gradient. The purified protein samples were stored at −20°C in 50% glycerol. The homogeneity of the protein preparations was verified by SDS-PAGE (Figure S1 in File S1).

### DNA repair assays

The standard reaction mixture (20 μl) used for screening of the wheat AP endonuclease-catalyzed DNA repair activities contained 10 nM ^32^P-labelled oligonucleotide substrate (THF•T, Exo20•G, Exo20^THF^•T, or Exo20^P^•T), 20 mM HEPES-KOH (pH 7.0), 50 mM KCl, 1 mM MnCl_2_, 1 mM DTT, 0.1 mg·ml^−1^ BSA, 0.1% Nonidet P-40 (NP-40) and 5 nM TaApe1L protein for 5 min at 23°C, unless specified otherwise.

The assay conditions for APE1 varied depending on the DNA repair pathways studied. The standard AP endonuclease (BER) assay was performed under high Mg^2+^ concentration (≥5 mM): the reaction mixture (20 μl) contained 10 nM ^32^P-labeled THF•T duplex oligonucleotide, 5 mM MgCl_2_, 50 mM KCl, 20 mM HEPES-KOH (pH 7.6), 0.1 mg•ml^−1^ BSA and 1 nM enzyme at 37°C for 5 min, unless specified otherwise. The standard NIR, exonuclease and 3′-repair phosphatase/phosphodiesterase assays were performed at a low Mg^2+^ concentration (≤1 mM) and acidic/neutral pH 6.8: the reaction mixture (20 μl) contained 10 nM ^32^P-labeled duplex oligonucleotide, 0.1 and 1.0 mM MgCl_2_ (for NIR and 3′-repair activities, respectively), 50 mM KCl, 20 mM HEPES-KOH (pH 6.9), 0.1 mg•ml^−1^ BSA and 1 nM APE1, unless specified otherwise. Assays were performed at 37°C for 10 min, unless specified otherwise.

To measure kinetics parameters, 10–300 nM of a duplex oligonucleotide substrate was incubated under the standard reaction conditions. The reaction products were separated and quantified using denaturing PAGE. For *K*_M_ and *k*_cat_ determination, the linear velocity was measured and the constants were determined with a one-site binding (hyperbola) model using Prism 4 (GraphPad Software). The kinetic parameters for exonuclease activity, when multiple degradation fragments appear, were determined by measuring the reaction products as integrated intensities of the fragments (expressed as percentage of total substrate). The value obtained for each fragment was multiplied by the number of catalytic events required for its formation, and the total exonuclease degradation was calculated as the sum of those products.

To measure kinetics parameters for TaApe1L-catalyzed 3′-phosphatase activity we used an indirect assay that can detect 3′-OH termini by DNA polymerase-catalyzed incorporation of a radioactive precursor. To do this, the recessed duplex Exo20^P^•G was incubated first with TaApe1L and then with DNA polymerase β in the presence of [α-^32^P]-3′-dATP (cordycepin). The reaction products were separated by denaturing gel electrophoresis and formation of the 3′-[α-^32^P]-labelled 21-mer Exo20-pA was measured. It was assumed that the amount of the labelled 21-mer product should be proportional to the amount of 3′-P removed by TaApe1L.

Assays for the Nth, Fpg and UDG proteins were performed in a buffer containing 20 mM HEPES-KOH (pH 7.6), 50 mM KCl, 1 mM EDTA, 1 mM DTT and 0.1 mg•ml^−1^ BSA. For the monofunctional DNA glycosylases, the abasic sites left after excision of damaged bases were cleaved by co-incubation with either the purified AP endonuclease or an AP lyase.

All reactions were stopped by adding 10 μl of a solution containing 0.5% SDS and 20 mM EDTA and then desalted by passing through house-made spin-down columns filled with Sephadex G25 (GE Healthcare) equilibrated in 7 M urea. Desalted reaction products were separated by electrophoresis in denaturing 20% (w/v) polyacrylamide gel (7 M urea, 0.5×TBE). Gels were exposed to a Fuji FLA-3000 Phosphor Screen and analyzed using Image Gauge v3.12 software.

### Preparation of anti-TaApe1L antibodies and Western blotting

About 1.4 mg of the purified recombinant TaApe1L protein was mixed with Freund's complete adjuvant and injected into rabbits. Three additional injections were made at 2-week intervals. One week after the last injection, the blood serum was collected and the production of anti-TaApe1L antibodies was checked by immunoblotting. Animal maintenance and experimental procedures were in accordance with the Kazakhstan national regulations based on the provisions of the Commission on Bioethics of the Research Center for Anti-Infectious Treatments (Almaty, Kazakhstan) and the Terms of Use of Laboratory Animals of the National Institutes of Health (USA). The Commission on Bioethics approved that the laboratory practice is in compliance with legal and ethical standards for the treatment of laboratory animals (Almaty, Kazakhstan protocol number 1571/13 from 14.03.2013).

Wheat (*T. aestivum*, variety Kazakhstanskaya 10) grains were sterilized in 2% (v/v) NaOCl for 20 min and washed twice with sterile water, once with 0.01 M HCl and then thoroughly with sterile distilled water. The grains were allowed to germinate at room temperature on sterile filter paper soaked in water in the presence of 5 μM gibberellic acid (GA_3_) for 4 days. Tissues were ground in liquid nitrogen and then resuspended in a buffer containing 50 mM Tris-HCl (pH 7.6), 2 mM DTT, 1 mM phenylmethylsulfonyl fluoride, 1 μg·ml^−1^ leupeptin, 1 μg·ml^−1^ pepstatin, 20 mM EGTA and 50 mM EDTA, the cell debris were pelleted, and the protein concentration was determined using the Bradford protein assay kit (Bio-Rad, France). Thirty five μg of the soluble protein were resolved by 10% (w/v) SDS-PAGE and transferred to a polyvinylidene difluoride membrane. For Western analysis, the membrane was blocked for 1 h in Tween 20/Tris-buffered saline (TBST, 50 mM Tris-HCl, pH 7.6, 150 mM NaCl, 0.1% [w/v] Tween 20) with 5% (w/v) reconstituted dry milk and incubated overnight at 4°C with the anti-TaApe1L rabbit polyclonal antiserum diluted 1∶500 in TBST. The blots were then washed with TBST, incubated with goat anti-rabbit horseradish peroxidase-conjugated antibody (Sigma) diluted 1∶10,000 (v/v) for 1 h, and TaApe1L was detected using chemilumensence (GE Healthcare).

### TaApe1L model building

The three-dimensional model of TaApe1L was built based on its homology to human APE1 using SWISS-MODEL homology modeling server [24]. Three structures of human APE1 were taken as templates: a 1.92-Å structure of APE1 bound to Mg^2+^ (PDB ID 4LND, chain A [25]), a 2.65-Å structure of APE1 bound to abasic DNA substrate in the absence of metal ions (1DEW, chain A [26]), and a 2.39-Å structure of APE1 bound to cleaved DNA product and Mg^2+^ (4IEM, chain A [27]). The target (TaApe1L) and the templates were aligned using Clustal Omega [28]), the target sequence was clipped to fully align with the template, and loops >3 amino acids long in the target were eliminated. All such loops were on the outer surface of the protein far away from the metal-binding sites; there were no loops in the aligned template sequence. The alignments and template PDB coordinates were submitted to the SWISS-MODEL server. The global quality of the models was assessed using QMEAN4 Z-score [29], and the local quality of the model around putative metal-binding sites was estimated using QMEAN, Anolea mean force potential [30]), and GROMOS empirical force field energy [31]. Models using either AtApe1L as a target or other protein chains in the PDB files as templates were prepared and analyzed in the same way.

## Results

### cDNA cloning, nucleotide sequence analysis and purification of the TaApe1L protein

**Figure 1:**
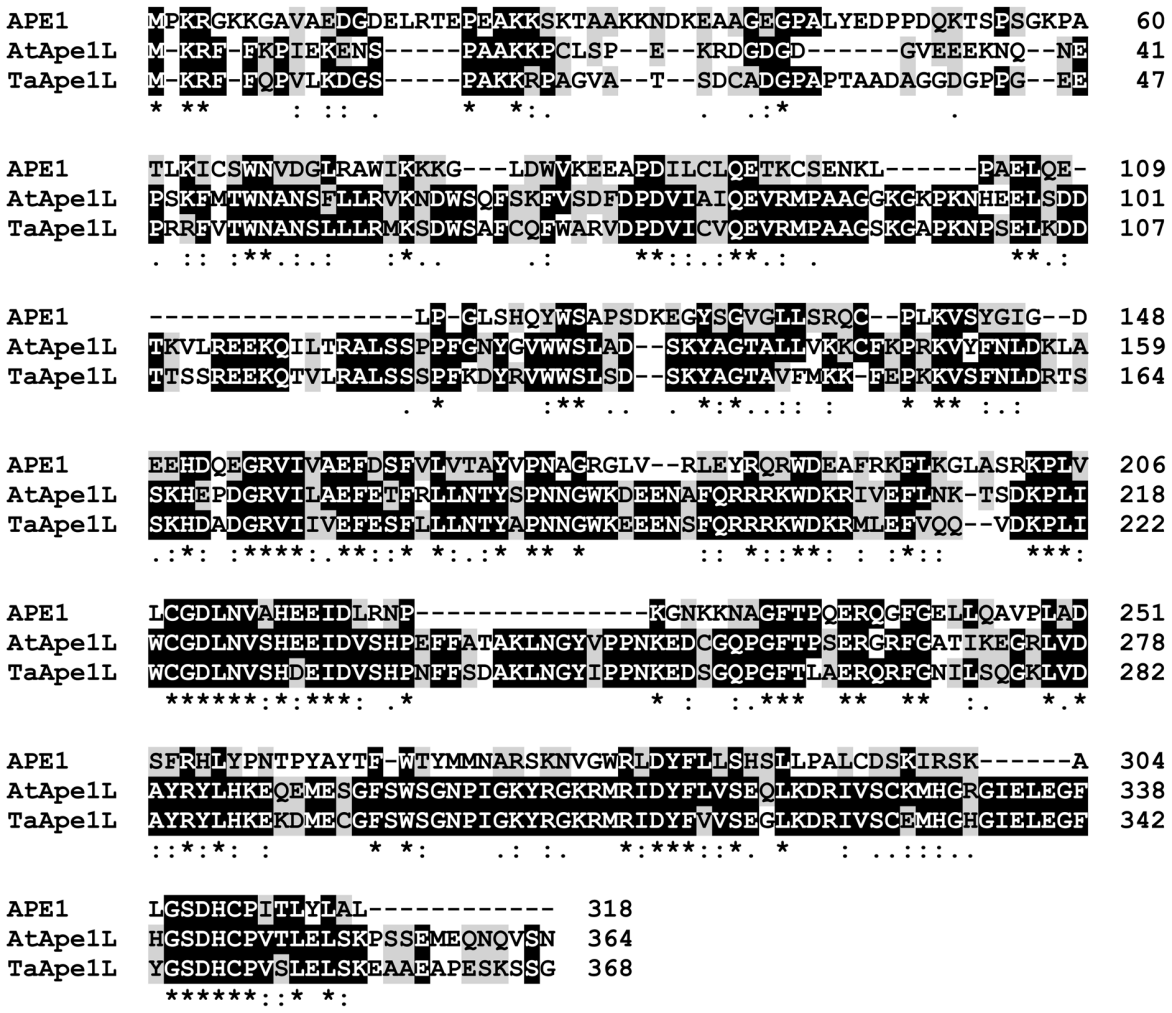
Protein sequence alignment of putative *T. aestivum* AP endonuclease (TaApe1L), putative *A. thaliana* AP endonuclease (AtApe1L), and human APE1. The deduced amino acid sequences were aligned using ClustalX 2.1. Asterisks (*), colons (:), and periods (.) indicate identical, conservative, and semi-conservative aligned residues, respectively.

Using the tBLASTn search we have identified a *T. aestivum* cDNA econding a putative AP endonuclease TaApe1L by its homology to the human APE1 and *A. thaliana* AtApe1L proteins. The TaApe1L protein sequence alignment showed high similarity with human APE1 (31% sequence identity) and *Arabidopsis* Ape1L (68% sequence identity) (Figure 1). The alignment of TaApe1L with the corresponding translated sequences of human APE1 and Arabidopsis AtApe1L, Arp and Ape2 indicates that the putative wheat protein belongs to the human APE1-like subfamily of the ExoIII family of apurinic/apyrimidinic endonucleases (Figure 1 and Figure S2 in File S1). Next, we isolated a cDNA encoding TaApe1L from wheat using RT-PCR. For this purpose, total RNA from the young leaves of *T. aestivum* variety Kazakhstanskaya 10 was extracted by trizole as described in the method section and used as template for first strand cDNA synthesis by RT-PCR with oligo-dT_18_ primer. The subsequent PCR using the first strand of cDNA as a template with the specific primers (see method section) yielded an approximately 1100-bp fragment which was ligated into the pBluescriptII SK(+) vector to form a pBluescriptII SK(+)/*taape* recombinant construct. The competent *E. coli* DH5α cells were transformed with the ligation product, and the plasmids isolated from the transformed colonies were sequenced. The cDNA insert in the pBluescriptII SK(+)/*taape* construct is 1116 bp long and contains a single open reading frame predicted to code for a protein of 368 amino acids. The calculated molecular mass of TaAPE1L is 41.3 kDa. Analysis of the primary amino acid sequence indicates that TaApe1L is rich in basic amino acids (Arg/Lys, 14.4%; pI = 7.47).

To characterize the DNA repair activities of TaApe1L, we have affinity-purified the wheat protein from *E. coli* Rosetta (DE3) strain expressing the His-tagged form of TaApe1L. Coomassie-stained gels revealed that the purified TaApe1L did not contain contaminating proteins and migrated slightly below the 50-kDa size marker (Figure S1 in File S1).

### The wheat AP endonuclease, TaApe1L cleaves duplex DNA containing a synthetic AP site

**Figure 2:**
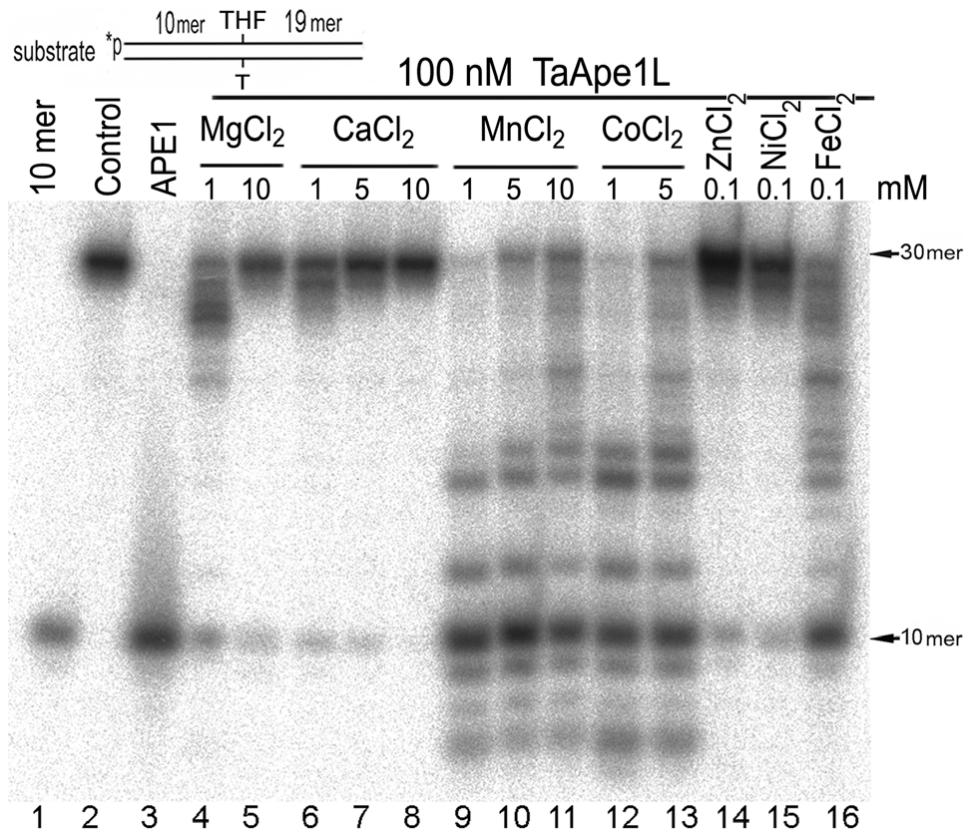
Divalent cation dependence of AP endonuclease activity of wheat TaApe1 protein on the oligonucleotide duplex THF•T. 10′-^32^P-labelled 30-mer DNA duplex containing a single THF residue was incubated for 5 min at 23°C with 100 nM TaApe1 under BER conditions but in the presence of different divalent cations. Lane 1, 10-mer size marker; lane 2, control non-treated 30-mer duplex THF•T; lane 3, as lane 2 but incubated with 1 nM APE1 for 5 min at 37°C; lane 4, as lane 2 but incubated with 100 nM TaApe1 and 1 mM MgCl_2_; lane 5, as lane 4 but in the presence of 10 mM MgCl_2_, lane 6, as lane 4 but in the presence of 1 mM CaCl_2_ instead of MgCl_2_, lane 7, as lane 4 but in the presence of 5 mM CaCl_2_ instead of MgCl_2_, lane 8, as lane 4 but in the presence of 10 mM CaCl_2_ instead of MgCl_2_, lane 9, as lane 4 but in the presence of 1 mM MnCl_2_ instead of MgCl_2_, lane 10, as lane 4 but in the presence of 5 mM MnCl_2_ instead of MgCl_2_, lane 11, as lane 4 but in the presence of 10 mM MnCl_2_ instead of MgCl_2_, lane 12, as lane 4 but in the presence of 1 mM CoCl_2_ instead of MgCl_2_, lane 13, as lane 4 but in the presence of 5 mM CoCl_2_ instead of MgCl_2_, lane 14, as lane 4 but in the presence of 0.1 mM ZnCl_2_ instead of MgCl_2_, lane 15, as lane 4 but in the presence of 0.1 mM NiCl_2_ instead of MgCl_2_, lane 16, as lane 4 but in the presence of 0.1 mM FeCl_2_ instead of MgCl_2_. For details, see Materials and Methods.

Previous studies demonstrated that ExoIII family AP endonucleases from *E. coli* (Xth), human (APE1) and plants (*A. thaliana* Arp) have an absolute requirement for divalent cations, typically Mg^2+^, for their catalytic activities [13], [32], [33]. Based on these observations, we measured the AP endonuclease activity of TaApe1L on a 5′-^32^P-labelled 30-mer oligonucleotide duplex THF•T under reaction conditions optimal for Xth and APE1 in a buffer supplemented with various divalent metal chlorides including MgCl_2_, CaCl_2_, MnCl_2_, CoCl_2_, ZnCl_2_, NiCl_2_ and FeCl_2_. As shown in Figure 2, in the presence of varying concentrations of Mg^2+^ and Ca^2+^, the purified TaApe1L protein taken in an excess (100 nM) exhibited only a weak AP endonuclease activity (lanes 4–8). Interestingly, higher concentrations of Mg^2+^ and Ca^2+^ (5–10 mM) resulted in strong decrease of the wheat enzyme-catalyzed AP site cleavage (lanes 5 and 8). In contrast, in the presence of 5 mM MgCl_2_ human APE1 cleaved the THF•T duplex completely (lane 3). At lower concentrations of Mg^2+^ and Ca^2+^, TaApe1L exhibited a robust 3′→5′ exonuclease activity (lanes 4 and 6) which was strongly suppressed when MgCl_2_ and CaCl_2_ concentrations were increased up to 10 mM (lanes 5 and 7–8). Examination of the effects of other divalent cations revealed that the presence of varying concentrations of MnCl_2_, CoCl_2_, or 0.1 mM FeCl_2_ greatly stimulated cleavage of AP site containing oligonucleotide duplex by TaApe1L (lanes 9–13 and 16). On the contrary, the presence of 0.1 mM ZnCl_2_ and NiCl_2_ in the reaction buffer did not stimulate the TaApe1L-catalyzed AP endonuclease activity (lanes 14 and 15). Interestingly, TaApe1L exhibited higher AP site cleavage activity in the presence of 1 mM Mn^2+^ and Co^2+^ as compared to higher (5–10 mM) concentrations of these cations (lanes 9 and 12 *versus* 10–11 and 13). To further substantiate the effects of divalent cations: Mg^2+^, Ca^2+^, Co^2+^, Zn^2+^, Ni^2+^ and Fe^2+^ on the TaApe1L-catalyzed activities, we incubated 5′-labelled THF•T duplex with limiting concentrations of the enzyme (5–10 nM) in the presence of 0.1% Nonidet P-40 for 5 min at 23°C. Under these conditions, TaApe1L exhibited mainly a non-specific 3′→5′ exonuclease activity and a very weak AP endonuclease activity, which were strongly stimulated in the presence of Co^2+^, Ni^2+^ and Fe^2+^ ions and to a lesser extent by Mg^2+^ but were strongly inhibited by Ca^2+^ and Zn^2+^ ions (Figure S3 in File S1). It should be noted that DTT was omitted from the buffer in the reactions with CoCl_2_ and NiCl_2_ to avoid reduction and precipitation of those metals, and that a higher concentration of TaApe1 (10 nM) was used to compensate for the absence of the reducing agent. Taken together, these results suggest that the wheat TaApe1L protein contains intrinsic strong 3′→5′ exonuclease and weak AP endonuclease activities and prefers Mn^2+^, Co^2+^, Ni^2+^ and Fe^2+^ cations as metal cofactors.

### Structural modelling and comparative analysis of human and wheat AP endonucleases

**Figure 3:**
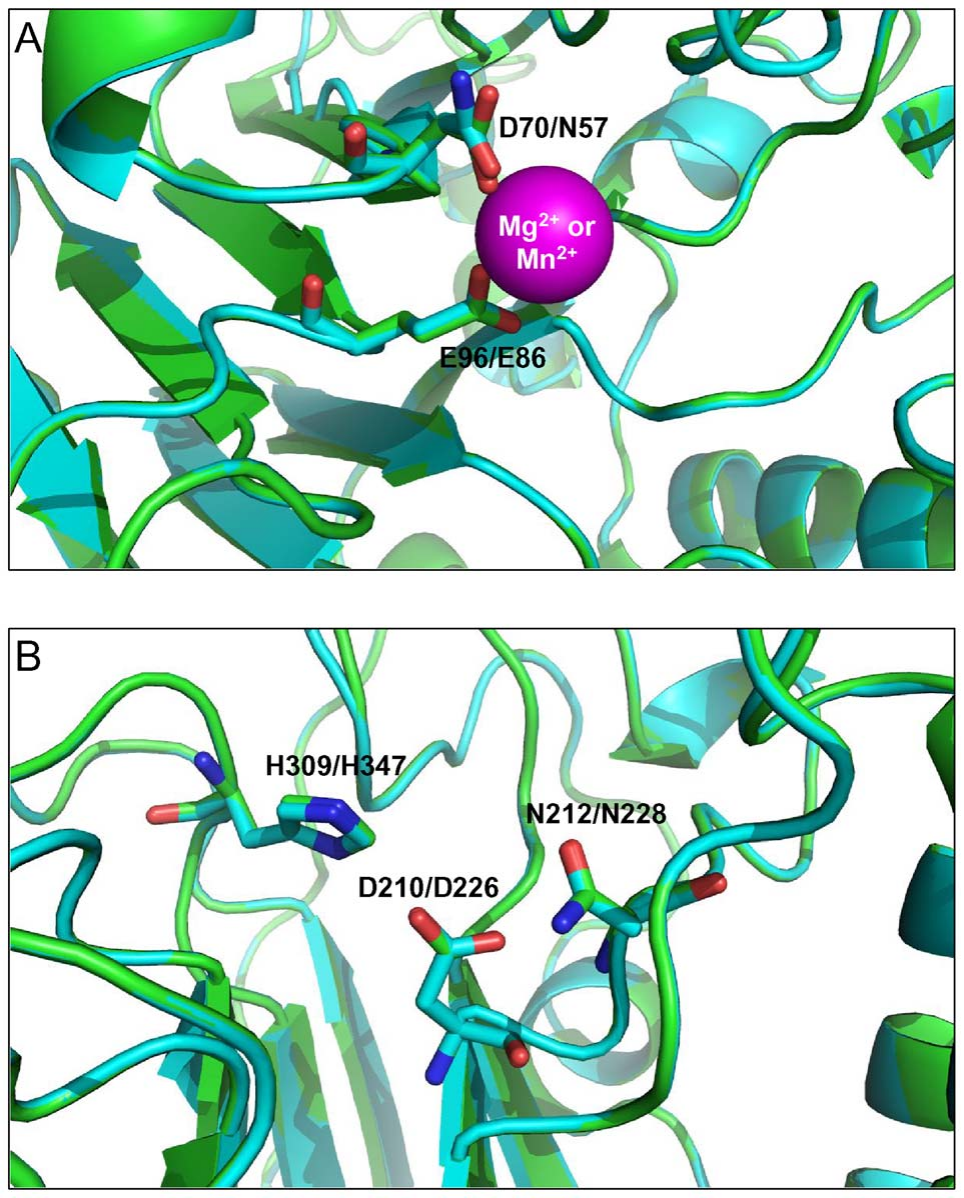
Alignment of human APE1 structure (4LND, the protein backbone and carbons are shown in green) and the homologous model of TaApe1L (the protein backbone and carbons are shown in cyan). In the amino acid labels, the first label always indicates the nature of the residue and its number in human APE1, the second one, in TaApe1L. The bound metal ion is shown as a magenta van der Waals sphere, the side chains of the protein ligands as sticks. A, metal-binding Site A. Note the difference between Asp in APE1 and Asn in TaApe1L. B, metal-binding site B showing an excellent overlap between the side chain ligands Asp, Asn, and His. The image was prepared using PyMol [44].

In order to rationalize the unusual preference for Mn^2+^, which is often interchangeable with Mg^2+^ in biological systems, we have modeled the three-dimensional structure of TaApe1L based on its homology to APE1, for which several crystal structures have been solved [25], [26], [27], [34], [35], [36] (Figure 3). Three structures of human APE1 representing different snapshots along the reaction pathway were taken as templates: APE1 bound to Mg^2+^ (4LND) [25], APE1 bound to abasic DNA in the absence of metal ions (1DEW) [26], and APE1 bound to cleaved DNA product and Mg^2+^ (4IEM) [27]. Of these, the model based on the apo protein (4LND) was the best based both on the global QMEAN4 score (0.62, Z-score −2.295, which puts the model quality within the population of experimental structures in the Protein Data Bank) and on the concordance of local estimators (QMEAN local score, Anolea mean force potential, GROMOS empirical force field energy) that all gave favorable conformational energy around the metal-binding sites. Therefore, this model will be discussed below, yet the principal findings were the same for all other models, both of TaApe1L and AtApe1L.

The structure of human APE1 contains two metal-binding sites. In Site A, the Mg^2+^ ion (or the replacement crystallographic Sm^3+^, Pt^2+^ and Pb^2+^ ions) has been observed in an octahedral co-ordination state, with two ligands belonging to the protein's side chains (Asp70 and Glu96) and four water molecules [25], [34], [35]. A lower-affinity Site B, involving Asp210, Asn 212, and His309, has been postulated based on the observance of a crystallographic Pb^2+^ ion and on the results of Ca^2+^ binding studies [35] but has not been seen populated by Mg^2+^ [25]. According to the accepted mechanism of APE1, if any metal is present in Site B, it should be displaced to allow the active site and DNA substrate to assume a catalytically competent conformation [26], [27].

The inspection of the sequence alignment (Figure 1) and model/template overlay (Figure 3A) immediately revealed that the protein ligands co-ordinating the divalent cation in APE1 and TaApe1L Site A are different: Asp70 in APE1 is Asn in TaApe1L. Although both Mg^2+^ and Mn^2+^ can be co-ordinated by both carboxylates and amides in protein structures, Mg^2+^ has a clear preference for carboxylates while Mn^2+^ is less biased between these two types of chemical moieties [37], [38]. This may be due to differences in the outer electronic shell structure (full 2*s* ^2^2*p* ^6^ in Mg^2+^, high-spin 3*d* ^5^ in Mn^2+^) or in chemical hardness of these ions (Mg^2+^ and Ca^2+^ are typical “hard Lewis acids” in terms of the HSAB theory and tend to react with “hard Lewis bases” such as carboxylate while Mn^2+^, Zn^2+^, Co^2+^, Ni^2+^ and Fe^2+^ are “softer” and tend to react with less electronegative, less oxidized, and more polarizable groups). Whatever the reason for this preference is, the substitution of Asn for Asp in TaApe1L makes the coordination sphere of Site A less favorable for Mg^2+^ and more favorable for Mn^2+^. At the same time, the residues in Site B overlap almost perfectly (Figure 3B). We suggest that such arrangement of the ligands makes Mg^2+^ and Ca^2+^ to bind preferentially at Site B where they act as reaction inhibitors rather than co-factors. On the contrary, the micro-equilibrium of binding for Mn^2+^ and other soft divalent cations may be shifted towards catalytic Site A carrying Asn instead of Asp. The consequence would be the metal preference observed for TaApe1L activity.

### Optimal reaction conditions for the TaApe1L-catalyzed AP endonuclease activity

**Figure 4:**
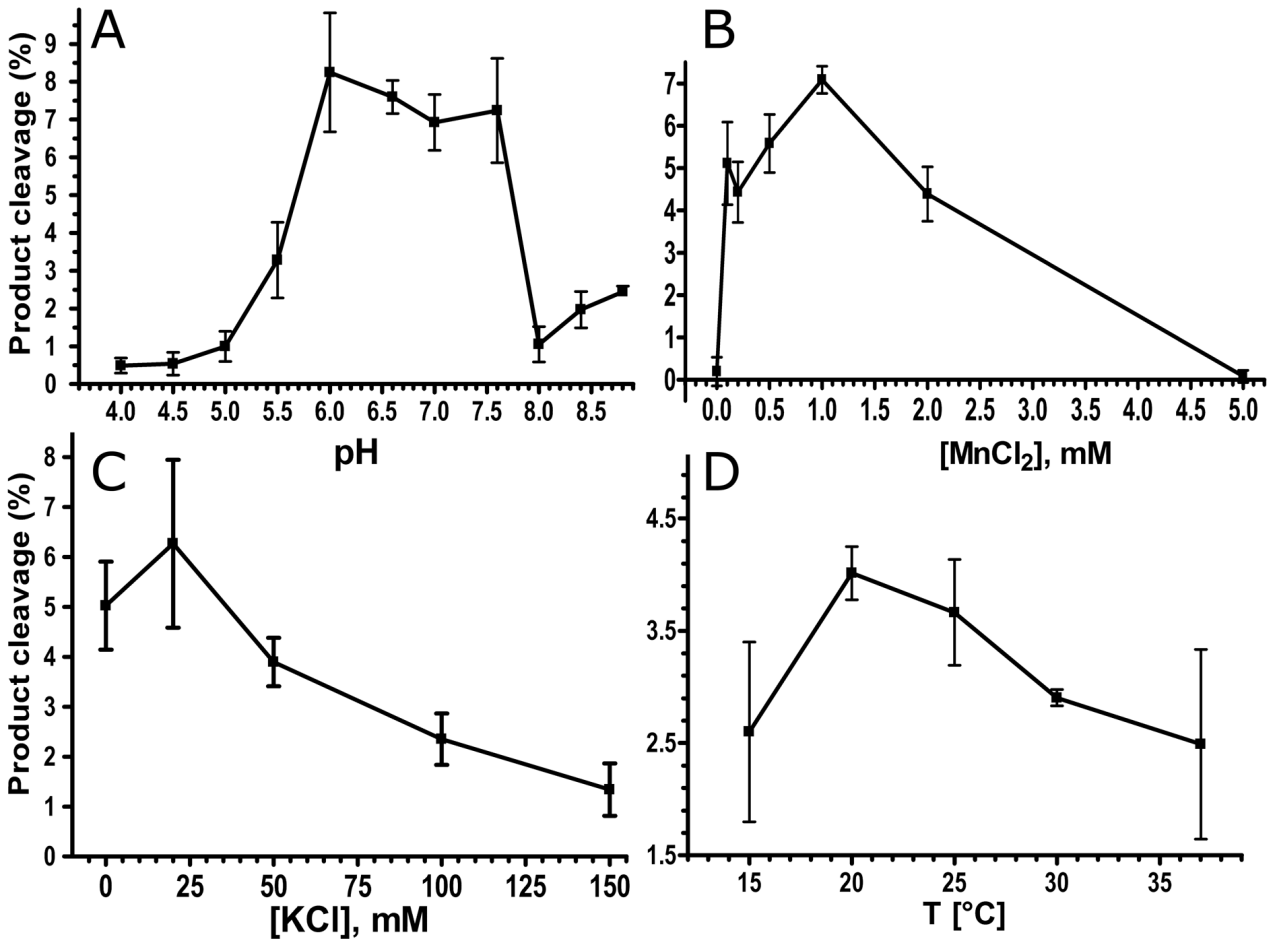
Dependence of the TaApe1L AP endonuclease activity on reaction conditions. (**A**) pH dependence, (**B**) concentration of Mn^2+^, (**C**) ionic strength, and (**D**) incubation temperature. For details see Materials and Methods.

**Figure 5:**
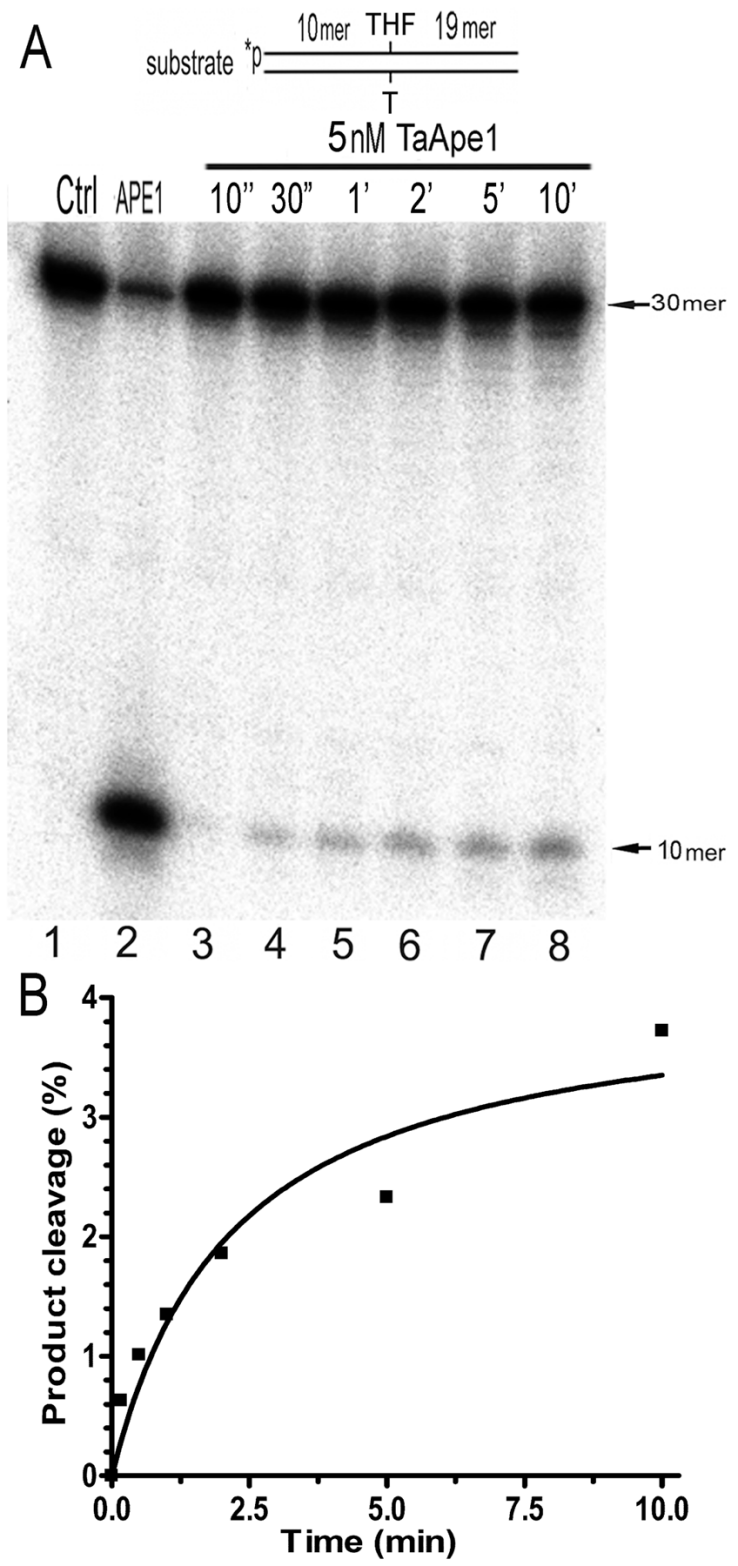
Time-dependent cleavage of the oligonucleotide duplex containing a synthetic AP site by TaApe1L. 10′-^32^P-labelled 30-mer TH•T was incubated with 5 nM TaApe1L at room temperature. (**A**) Separation of the reaction products by denaturing PAGE. (**B**) Graphical representation of time course of the TaApe1L-catalyzed cleavage of THF•T. For details, see Materials and Methods.

To characterise the biochemical properties of the AP site cleavage activity of TaApe1L, we measured the wheat enzyme-mediated cleavage of THF•T varying the concentration of MnCl_2_, ionic strength, pH and temperature. Importantly, we found a dramatic loss of the wheat enzyme-catalyzed activities after a 5–10-fold protein dilution in the reaction buffer; however, when the buffer was supplemented with 0.1% NP40 detergent, TaApe1L exhibited a robust activity even when diluted to a 1 nM concentration. As shown in Figure 4A–C, the pH-, ionic strength- and Mn^2+^-dependence of the TaApe1L-catalyzed abasic site cleavage activity generally exhibits a bell-shaped curve as a function of the reaction conditions. Interestingly, we observed a higher AP endonuclease activity of the wheat enzyme when incubating the mixture at 20°C as compared to higher temperatures (25°–37°C, Figure 4D). Based on these observations, we had established optimal standard reaction conditions for TaApe1, namely 1 mM MnCl_2_, 50 mM KCl, pH 7.0, and incubation at 23°C. As shown in Figure 5, we measured time-dependent cleavage of THF•T by TaApe1L under these reaction conditions. As expected, TaApe1L-catalyzed AP site cleavage increased with incubation time. The linear phase of time-dependent cleavage was observed only up to ∼2 min of incubation (Figure 5).

In the experiments described above we noticed that the wheat AP endonuclease is able to cleave AP site DNA even in the absence of any metal cofactors (Figure 4B). To examine the metal binding properties of TaApe1L, we measured the cleavage of the THF•T duplex in the presence of EDTA. An addition of 1 mM EDTA to the reaction mixture completely blocked AP endonuclease activity of TaApe1L (Figure S4 in File S1).

### Wheat TaApe1L possesses 3′-repair phosphatase and phosphodiesterase activities but not the NIR function

**Figure 6:**
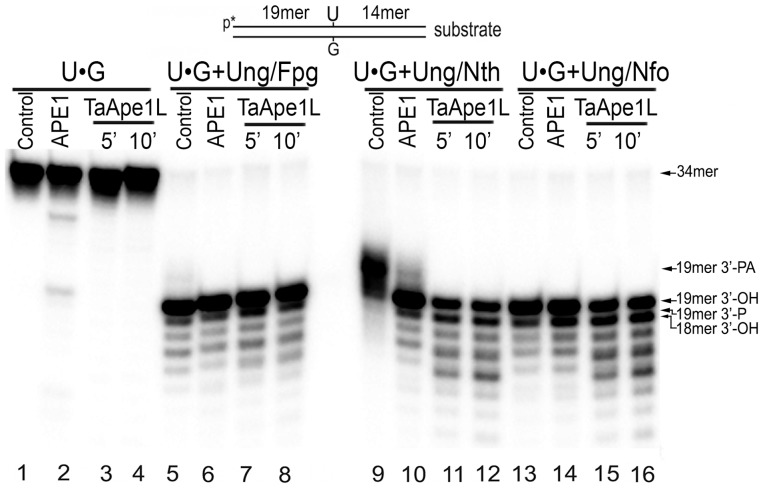
DNA repair activities of TaApe1L on gapped oligonucleotide duplexes containing 3′-P, 3′-PA and 3′-OH termini. 10′-^32^P-labelled 34-mer U•G duplex was treated first with hUNG/Fpg, hUNG/Nth or hUNG/Nfo and then incubated with either 5 nM APE1 at 37°C or with TaApe1L at room temperature under the respective optimal reaction conditions. For details, see Materials and Methods.

Many hydrolytic AP endonucleases, in addition to their AP site cleavage function, possess highly efficient activities to remove 3′-blocking phosphates or sugar-phosphate remnants from DNA strand breaks generated by oxidative damage or DNA glycosylases/AP lyases. To examine whether wheat AP endonuclease possesses such 3′-repair phosphatase or phosphodiesterase functions, we prepared DNA substrates containing a strand break or an 1-nt gap flanked with 3′-blocking termini of different nature. To achieve this, the 5′-^32^P-labelled 34-mer oligonucleotide duplex containing a single U•G base pair was incubated with human uracil-DNA glycosylase (hUNG) to generate an AP site. Then, the resulting AP•G duplex was treated either by *E. coli* Fpg (a bi-functional DNA glycosylase/AP lyase cleaving the AP site by combined β/δ elimination) or by *E. coli* Nth (bi-functional DNA glycosylase/AP lyase cleaving only by β elimination) or by *E. coli* Nfo (hydrolytic AP endonuclease). These enzymes cleave DNA at an AP site and generate, respectively: a 1-nt gap flanked with a 3′-phosphate (3′-P), a single-strand break flanked with a 3′-α,β-unsaturated aldehyde (3′-PA) and a single-strand break flanked with a 3′-hydroxyl (3′-OH) groups, respectively (Figure S5 in File S1). As shown in Figure 6, incubation of U•G duplex with hUNG and bacterial AP lyases generates a 19-mer 5′-^32^P-labelled cleavage fragment that contains 3′-P (lane 5) and moves faster than the fragment with 3′-OH (lane 13), and a fragment with 3′-PA (lane 9) that moves much slower than other cleavage fragments on the denaturing PAGE. As expected, an incubation of the 5′-^32^P-labelled 19-mer cleavage fragments containing 3′-P and 3′-PA groups with human APE1 resulted in a change in their mobility indicating the removal of the 3′-blocking groups and generation of a 3′-OH group (lanes 6 and 10). Incubation of the 19-mer cleavage fragments containing 3′-P and 3′-PA groups with TaApe1L also generated fragments with a 3′-OH group (lanes 7–8 and 11–12). It should be noted that the 19-mer fragment with a 3′-P terminus (lane 5) migrated faster in the denaturing gel than the 3′-dephosphorylated 19-mer product (lanes 6–8) due to an additional negative charge of the phosphate group. When acting upon the 19-mer fragment containing a 3′-OH group, both APE1 and TaApe1L remove normal nucleotides in a non-specific manner by their intrinsic 3′→5′ exonuclease activitiy (lanes 14 and 15–16). Interestingly, removal of the 3′-PA group stimulated the 3′-exonuclease activity of TaApe1L (lanes 11–12) but not that of APE1 (lane 10). Taken together, these results suggest that the wheat TaApe1L contains robust 3′-end cleansing activities similar to its human homologue.

We further examined the DNA substrate specificity of wheat AP endonuclease by using duplex oligonucleotides containing alpha-anomeric 2′-deoxyadenosine (αdA) or 5,6-dihydrouracil (DHU). No cleavage of αdA•T and DHU•G duplexes was detected when they were incubated with the TaApe1L protein. In contrast, human APE1 incised the DNA duplexes 5′ to the lesion as expected (Figure S6 in File S1). These results indicate that wheat TaApe1L lacks any NIR activity under the experimental conditions tested.

### Steady-state kinetic characterization of the wheat AP endonuclease

To further characterize the DNA substrate specificity of the recombinant TaApe1L protein, we measured steady-state kinetic parameters of the repair reactions and calculated the *K*_M_, *k*_cat_, and *k*_cat_/*K*_M_ values for the cleavage of various DNA substrates by the wheat enzyme (Table 1). Recessed Exo20•G^REC^ and Exo20^P^•G^REC^ duplex oligonucleotides with 3′-OH and 3′-THF termini, respectively, were used to measure 3′→5′ exonuclease and 3′-phosphodiesterase activities, respectively. To measure the 3′-phosphatase activity we used a recessed oligonucleotide duplex Exo20^P^•G^REC^ containing a phosphate at the 3′-end of the shorter fragment. The wheat TaApe1L protein possesses an extremely weak AP endonuclease activity as compared to human APE1. Under optimal reaction conditions and limited enzyme concentration TaApe1L cleaved at most 4–8% of AP sites (Figures 4 and 5). Importantly, a further increase in the incubation time and enzyme concentration resulted in the extensive 3′→5′ exonucleolytic degradation of THF•T duplex before the AP site cleavage by TaApe1L exceeded 10%. This non-specific activity of the wheat enzyme obstructs accurate measurement of the amount of cleaved AP sites. In contrast to the AP site cleavage activity, wheat AP endonuclease showed robust 3′-phosphodiesterase, 3′-phosphatase and 3′→5′ exonuclease activities comparable to that of human APE1. Interestingly, the *k*_cat_/*K*_M_ value of TaApe1L for 3′-phosphatase activity is 2.5-fold higher than that of the human counterpart suggesting that the wheat enzyme removes 3′-P more efficiently than APE1 does (Table 1). Both wheat and human AP endonucleases contain robust 3′-repair phospodiesterase activity and remove the 3′-THF group with a similar catalytic efficiency. APE1, under the exonuclease reaction conditions, exhibits about 3-fold higher *k*_cat_/*K*_M_ value for the 3′→5′ exonucleolytic activity as compared to that of TaApe1L, suggesting that the human enzyme possesses a more efficient exonuclease functionality.

**Table 1:** Comparison of kinetic parameters of AP endonucleases from human and wheat.

Proteins	Human APE1^b^			Wheat TaApe1L			*k*_cat_/*K*_M_ ratio, APE1/TaApe1L
DNA substrate^a^	*K*_M_, nM	*k*_cat_, min^−1^	*k*_cat_/*K*_M_, min^−1^·μM^−1^	*K*_M_, nM	*k*_cat_, min^−1^	*k*_cat_/*K*_M_, min^−1^·μM^−1^	
THF•T	0.87	15	17200	N.D.^c^	N.D.	N.D.	-
Exo20^THF^•G	8.2	6.4	780	127	80	630	1.2
Exo20^P^•G	20	3.6	180	143	69	485	0.4
Exo20•G	2.4	0.86	360	270	36	132	2.7

a Each type of DNA substrates were used to measure a specific AP endonuclease activity under the appropriate optimal reaction conditions: THF•T for AP endonuclease activity, Exo20^THF^•G for 3′-repair phosphodiesterase activity, Exo20^P^•G for 3′-phosphatase activity and Exo20•G for 3′→5′ exonuclease activity (see *Materials and Methods*).

b APE1-catalyzed AP site cleavage and 3′→5′ exonuclease activities were measured under BER and EXO conditions, respectively. All data taken from [45].

c N.D.  =  not determined. Kinetic parameters could not be determined because of low enzymatic activity of TaApe1L.

Overall, the results suggest that wheat TaApe1L is an inefficient AP endonuclease and its role in the repair of AP sites *in vivo* may be limited. However, TaApe1L contains robust 3′-repair phosphodiesterase and 3′-phosphatase activities suggesting that it might play an important role in the repair of DNA glycosylase- and ROS-induced DNA strand breaks containing 3′-blocking groups *in vivo*.

### Drug sensitivity of *E. coli* AP endonuclease-deficient strains expressing TaApe1L and the presence of TaApe1L in wheat tissues

Previously it has been shown that the expression of human APE1 in an AP endonuclease-deficient *E. coli* complemented the hypersensitivity of this strain to alkylating agents but not to oxidizing ones such as H_2_O_2_, bleomycin and *tert*-butyl hydroperoxide [39]. Therefore, to address the physiological relevance of TaApe1L-catalyzed DNA repair activities, we used the *E. coli* mutant phenotype rescue assay. We examined the methylmethanesulfonate (MMS) and H_2_O_2_ sensitivity of the AP endonuclease-deficient *E. coli xth nfo* BH110 strain harboring a plasmid coding for the *E. coli* Nfo or wheat TaApe1L proteins. MMS, an alkylating agent that methylates DNA bases, indirectly generates AP sites in DNA when methylated purines are excised by DNA glycosylases in the BER pathway [40]. H_2_O_2_ causes oxidation of DNA bases as well as single-strand breaks containing 3′-blocking sugar-phosphate groups [41]. The *E. coli* BH110 strain lacking Xth and Nfo AP endonucleases is extremely sensitive to both agents [42]. As expected, the plasmid that directs the synthesis of Nfo conferred resistance to MMS and H_2_O_2_ on BH110 cells. In contrast, neither the control empty vector nor a plasmid encoding TaApe1L restored resistance to MMS or H_2_O_2_ (data not shown). These results suggest that both the very low AP endonuclease activity and the requirement for specific reaction conditions may contribute to the inefficient rescue of *E. coli xth nfo* grown on the media containing these genotoxic agents.

**Figure 7:**
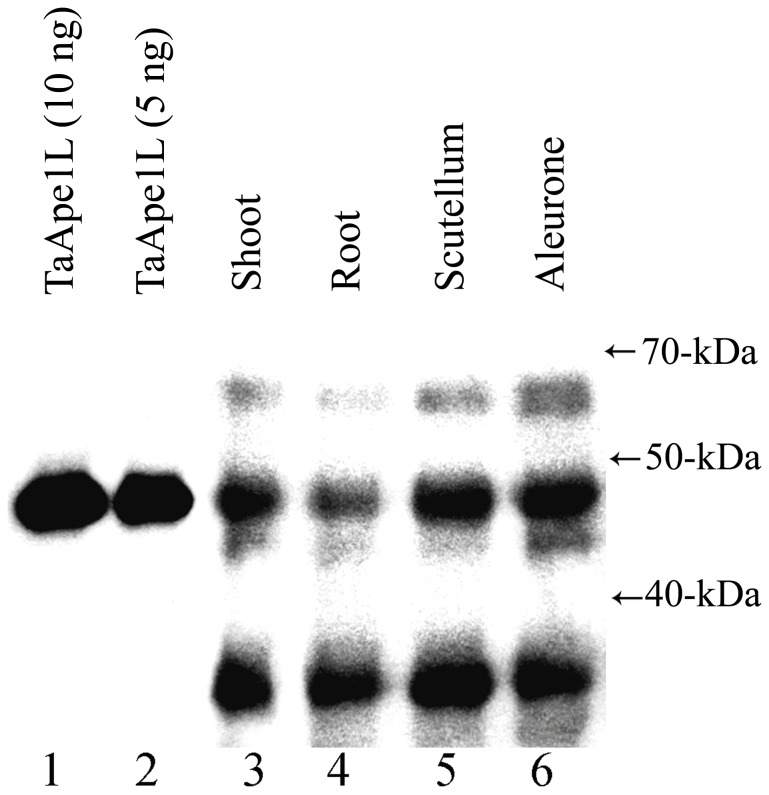
Presence of the TaApe1L protein in wheat seedling tissues. Wheat seedlings were grown for 4 days and then dissected into shoot, root, aleurone and scutellum tissues. 35 μg of soluble protein extracted from each tissue were separated using 10% SDS-PAGE, transferred to the membrane, and incubated with rabbit anti-TaApe1L polyclonal antiserum. Lane 1, 10 ng of the recombinant TaApe1L protein; lane 2, 5 ng of TaApe1L; protein extracts from shoot (lane 3), root (lane 4), scutellum (lane 5), and aleurone (lane 6). For details, see Materials and Methods.

Next, to examine whether TaApe1L is present during seedling growth, wheat seeds were germinated for four days, the seedlings were dissected, and the presence of TaApe1L was determined by Western blot analysis using rabbit polyclonal antibodies generated against the recombinant TaApe1L protein. The purified TaApe1L protein was used as a control in the Western blots. As shown in Figure 7, anti-TaAPE1 antibodies detect the recombinant TaApe1L protein as a single band migrating slightly below the 50-kDa size marker (lanes 1-2). In all tissue extracts from the phytohormone GA_3_-treated four-days-old seedlings, the antibodies detected a protein band at ∼50 kDa that co-migrates with the recombinant TaApe1L and also two other bands migrating below 70-kDa and 40-kDa size markers, respectively (lanes 3–6). These results suggest that the TaApe1L protein is present in all tissues of 4 days seedlings, at approximately equal levels in the aleurone and scutellum (lanes 5–6) and at a somewhat lower level in the shoot and roots (lanes 3–4).

## Discussion

The tBLASTn search for homologues of human APE1 in *Triticum aestivum* cDNA databases revealed the presence of a DNA clone encoding for a putative AP endonuclease with a close homology to the *Arabidopsis thaliana* AtApe1L protein. We have isolated cDNA encoding the putative wheat AP endonuclease, referred here as TaApe1L, and characterized its protein product. Our results show for the first time that the purified TaApe1L protein contains at least the following DNA repair activities: AP endonuclease, 3′-repair phosphodiesterase, 3′-phosphatase and 3′→5′ exonuclease. Importantly, TaApe1L lacks any NIR activity on DNA substrates containing oxidatively damaged bases, suggesting that other Arp-like and Ape2-like AP endonucleases may catalyze NIR activity observed previously in the extracts of wheat aleurone cells [20]. Interestingly, the wheat enzyme requires specific reaction conditions. In contrast to other known ExoIII family AP endonucleases from *E. coli*, yeast and human, TaApe1L-catalyzed AP endonuclease activity requires Mn^2+^ and is inhibited in the presence of Mg^2+^ cations. Furthermore, the wheat enzyme exhibits the highest activity at 20°C and is slightly inhibited at 37°C. We have established that the optimal reaction conditions for TaApe1L comprise low pH, low ionic strength, low concentration of divalent cations and ambient incubation temperature. It should be noted that these conditions are quite different from those optimal for *Arabidopsis* Arp and the well-studied *E. coli* Xth and human APE1 proteins. Under these conditions, TaApe1L exhibits robust 3′-repair phosphodiesterase, 3′-phosphatase and 3′→5′ exonuclease activities on nicked or gapped DNA duplexes containing 3′-blocking termini. It should be noted that the presence of a non-ionic detergent in the buffer is necessary when preparing serial dilutions in order to stabilize the protein and keep it active. This may suggest that the conformational stability of TaApe1L is quite weak and it tends to aggregate or denature at low protein concentration. Altogether these results indicate that DNA repair assays in plant cell-free extracts and *in vitro* reconstitution of the BER pathway using purified plant proteins should take in account the specific requirements for the Ape1L-like proteins and possibly for Ape2-like ones.

The analysis of kinetic parameters for the TaApe1L protein shows that the enzyme contains efficient 3′-repair phosphodiesterase, 3′-phosphatase and 3′→5′ exonuclease activities with *k*_cat_/*K*_M_ values comparable to that of human APE1 (Table 1). Surprisingly, the wheat enzyme cleaved oligonucleotide duplexes containing a synthetic AP site very poorly, making impossible accurate measurements of the kinetic parameters of AP endonuclease activity (Table 1). Efficient 3′-repair activities of the wheat AP endonuclease suggest that TaApe1L may play a role in 3′-end cleansing during both single-strand DNA break repair and DNA glycosylase/AP lyase-initiated BER pathways. Consistent with these biochemical data, expression of TaApe1L in *E. coli* AP endonuclease-deficient BH110 strain did not confer resistance to MMS and H_2_O_2_, suggesting that both the extremely weak AP endonuclease activity of the wheat enzyme and its requirement for the specific reaction conditions may contribute to the inefficient rescue of *E. coli* BH110 grown on the genotoxic media. Finally, taking together the previous observations on *Arabidopsis* and our biochemical data, we propose that in the absence of a Mg^2+^-dependent AP endonuclease, TaApe1L together with an AP lyase can repair AP sites in DNA through β-elimination of the phosphodiester backbone 3′ next to the AP site and subsequent removal of the resulting 3′-PA group to generate a 1-nt gap flanked with 3′-OH and 5′-P termini.

Western blot analysis of various wheat plant tissues obtained from germinating seeds using rabbit polyclonal anti-TaApe1L antibodies revealed the presence of the TaApe1L protein in all tissues examined (Figure 7). These results suggest that in germinating wheat, TaApe1L may function in the BER pathway to protect genome integrity in dividing and differentiated cells. The genome of *A. thaliana*, encodes three AP endonucleases homologous to human APE1: Arp, Ape1L and Ape2. Although the search of *T. aestivum* DNA sequence data bank identified only one AP endonuclease TaApe1L, it is very likely that the wheat genome also contains two other AP endonucleases homologous to *Arabidopsis* Arp and Ape2. To examine this possibility we performed a BLAST search for homologues of human APE1 in the fully sequenced genome of rice (*Oryza sativa*), a cereal related to wheat more closely than the eudicot *Arabidopsis*. As expected, we have identified rice homologues of Arp (GenBank: BAD68138.1), Ape1L (Gene ID: 4351982) and Ape2 (Gene ID: 4347697). These results strongly suggest that the wheat genome, similar to the genome of rice, contains, in addition to TaApe1L, two other putative AP endonucleases homologous to *Arabidopsis* Arp and Ape2. The presence of at least three putative AP endonucleases in cereals indicate a functional redundancy in the repair of AP sites and 3′-blocking groups in *Poaceae*. In *Arabidopsis*, Arp protein is responsible for the major AP endonuclease activity in extracts, and mutant plants deficient in *Arp* are hypersensitive to DNA damage. We speculate that a very weak AP site cleavage activity of TaApe1L is due to the presence of other wheat AP endonuclease homologs that may contain a highly efficient AP endonuclease function. Mechanistically, the preference of TaApe1L for 3′-end cleansing rather than AP endonuclease activity may arise from differences in the active site structure from typical AP endonucleases of the ExoIII family. In human APE1, residues Phe266 and Trp280 form part of the binding pocket where the AP site falls after its eversion from the double helix [26]. Site-directed mutagenesis and structural studies in another member of the ExoIII family, exonuclease III from *Archaeoglobus fulgidus*, show that elimination of aromatic residues at these positions leads to an increase in exonucleolytic activities at the expense of AP endonuclease [43]. In TaApe1L, the Phe residue, which helps to extrude the target nucleotide, is conserved, but the Trp residue is replaced with Met (Figure 1). This would likely make the pocket wider, possibly suboptimal for tight binding of an internal AP site but suitable for accommodation of a variety of 3′-terminal moieties. It should be noted that *E. coli* exonuclease III, a prototypical member of the ExoIII family, has non-aromatic Leu at this position and possesses strong 3′-terminal processing activities.

Previously, a study of the phenotypes of knock-out AP endonuclease mutants of *A. thaliana* demonstrated that *Ape1L* and *Ape2*, but not *Arp*, are essential for the development of the embryo suggesting that the AP endonuclease-dependent BER pathway is required for seed development [17]. Recently, Roldán-Arjona and colleagues using *in vitro* reconstitution assays of the 5mC-DNA glycosylase/AP lyase-initiated active DNA demethylation in *Arabidopsis* revealed the existence of two alternative pathways for cleansing 3′-ends generated by ROS1: ZDP-dependent and ZDP-independent [16]. They have demonstrated that ZDP is the main 3′-phosphatase activity in *Arabidopsis* cell-free extracts required for the processing of 3′-P termini generated by ROS1. The authors also demonstrated the presence of an unknown 3′-repair phosphodiesterase activity in cell-free extracts that removes 3′-PA groups and other DNA demethylation intermediates generated by ROS1 in an ZDP- and Arp-independent manner [16]. Our biochemical data show that the purified wheat TaApe1L contains robust 3′-repair phosphodiesterase and 3′-phosphatase activities but has an extremely weak AP endonuclease function. The fact that AtApe1L can substitute for Arp and Ape2 in *A. thaliana Arp^–/–^*, *Ape2^–/–^* double mutant indicate that this AP endonuclease can repair both AP sites and 3′-blocking groups *in vivo*. We propose that in wheat and *Arabidopsis*, Ape1L and possibly Ape2 both participate in the 3′-end cleansing of DNA strand breaks generated during active DNA demethylation. Consequently, Ape1L and Ape2 are essential for the completion of active DNA demethylation occurring during embryonic development in plants.

In conclusion, we have biochemically characterized the activities of TaApe1L, a member of ExoIII family of AP endonucleases from wheat. Since TaApe1L demonstrated a low AP endonuclease activity *per se* but had robust 3′-end cleansing activities, we refrain from definitely assigning this enzyme to the BER pathway and note that additional data are required to predict with more certainty its functional implications *in vivo*. At present very scarce literature data available on the BER pathway in cereals. Future studies of biochemistry and genetics of wheat BER proteins, particularly of orthologs of *Arabidopsis* AP endonucleases and DNA glycosylases specific for various kinds of modified nucleobases, will clarify the features of DNA damage processing and its interplay with DNA demethylation and seed development in this highly important group of plants.

## Supporting Information

File S1 **Figures S1–S6.** Figure S1. SDS-PAGE analysis of the purified recombinant TaApe1 protein. Lane 1, protein size markers; lane 2, human APE1, 2 μg; lane 3, TaApe1, 1 μg; lane 4, TaApe1, 5 μg. Figure S2. Protein sequence alignment of human APE1, *Arabidopsis thaliana* AP endonucleases AtApe1L, AtArp and AtApe2. The deduced amino acid sequences were aligned using ClustalX 2.1. Asterisks (*), colons (:), and periods (.) indicate identical, conservative, and semi-conservative aligned residues, respectively. Figure S3. Divalent cation dependence of wheat TaApe1-catalyzed activities on the oligonucleotide duplex THF•T. 10 nM 5′-^32^P-labelled 30-mer DNA duplex containing a single THF residue was incubated for 10 min at 23°C with 5 or 10 nM TaApe1 under standard reaction conditions but in the presence of different divalent cations. (A) Effects of MgCl_2_, CaCl_2_ and CoCl_2_ on the enzyme activities (B) Effects of ZnCl_2_, NiCl_2_ and FeCl_2_ on the enzyme activities. Lanes 1, 3, 7, 11, control 30-mer duplex THF•T incubated in reaction buffer without enzyme; lane 2, as lane 1 but incubated with 1 nM APE1 for 5 min at 37°C; lanes 4–6, 8–10 and 12–14, 30-mer duplex THF•T incubated with TaApe1L in the presence of the indicated metal chloride. For details, see Materials and Methods. Figure S4. Effects of the absence of divalent cations and/or presence of 1 mM EDTA in the reaction buffer on the TaApe1L AP endonuclease activity. 10 nM 5′-^32^P-labelled 30-mer DNA duplex containing a single THF residue was incubated with 5 nM TaApe1L at 23°C. Lane 1, control non-treated THF•T duplex; lane 2, as lane 1 but incubated for 5 min at 37°C with 1 nM APE1; lane 3, as lane 2 but incubated with 5 nM TaApe1L and 1 mM MnCl_2_ for 5 min at 23°C; lane 4, as lane 3 but incubated with 10 nM TaApe1L: lane 5, as lane 3 but incubated with 0 mM MnCl_2_; lane 6, as lane 5 but incubated with 10 nM TaApe1L; lane 7, as lane 3 but incubated with 1 mM EDTA; lane 8, as lane 7 but incubated with 10 nM TaApe1; lane 9, as lane 3 but incubated with 5 mM EDTA; lane 10, as lane 9 but incubated with 10 nM TaApe1. The arrows mark the position of the 30-mer DNA substrate and 10-mer cleavage product. For details, see Materials and Methods. Figure S5. Preparation of the DNA substrates containing a nick flanked with 3′-PA or 3′-OH termini or a 1-nt gap flanked with 3′-P to measure 3′-repair phoshodiesterase activities of TaApe1L. (A) Schematic representation of the structures of 3′ and 5′ DNA termini at a DNA nick or gap. (B) Separation of 5′-^32^P-labelled oligonucleotide fragments by denaturing PAGE. 10 nM 5′-^32^P-labelled 34-mer DNA duplex containing a single U•G base pair was incubated for 5 min at 37°C with 10 nM hUNG and then with either 20 nM Fpg or 20 nM Nth or 20 nM Nfo for 15 min at 37°C. Lane 1, control non-treated 34-mer duplex U•G; lane 2, as lane 1 but incubated with 10 nM hUNG and 20 nM Fpg; lane 3, as lane 1 but incubated with 10 nM hUNG and 20 nM Nth; lane 4, as lane 1 but incubated with 10 nM hUNG and 20 nM Nfo. The arrows mark the position of the full-length 34-mer substrate and 19-mer cleavage fragments containing by 3′-PA, 3′-OH and 3′-P. For details, see Materials and Methods. Figure S6. Activities of human APE1 and TaApe1L on αdA•T and DHU•G oligonucleotide duplexes. 10 nM 5′-^32^P-labelled 30-mer DNA duplex containing a single modified residue was incubated with 5 nM TaApe1L at 23°C. Lane 1, control non-treated THF•T duplex; lane 2, as lane 1 but treated with 1 nM APE1 for 5 min at 37°C; lane 3, as lane 1 but treated with 5 nM TaApe1L for 5 min; lane 4, as lane 3 but treated for 10 min; lane 5, as lane 3 but treated for 15 min; lane 6, as lane 3 but treated for 30 min; lane 7, control non-treated αdA•T duplex; lane 8, as lane 7 but treated with 5 nM APE1 for 5 min at 37 °C; lane 9, as lane 7 but treated with 5 nM TaApe1L for 5 min; lane 10, as lane 9 but treated for 10 min, lane 11, as lane 9 but treated for 15 min, lane 12, as lane 9 but treated for 30 min; lane 13, control non-treated DHU•G duplex; lane 14, as lane 13 but treated with 5 nM APE1 for 5 min at 37°C; lane 15, as lane 13 but treated with 5 nM TaApe1L for treated for 5 min; lane 16, as lane 15 but treated for 10 min, lane 17, as lane 15 but treated for 15 min, lane 18, as lane 15 but treated for 30 min. The arrows mark the position of the full-length 30-mer substrate and 10-mer AP-endonuclease and NIR cleavage products. For details, see Materials and Methods.
(PDF)
