# Correction: Cloning and Characterization of a Wheat Homologue of Apurinic/Apyrimidinic Endonuclease Ape1L

**DOI:** 10.1371/journal.pone.0101795

**Published:** 2014-06-26

**Authors:** The PLOS ONE Staff

The fifth author’s name is spelled incorrectly. The correct name is: Alexander Ischchenko. The correct citation is: Joldybayeva B, Prorok P, Grin IR, Zharkov DO, Ishchenko AA, et al. (2014) Cloning and Characterization of a Wheat Homologue of Apurinic/Apyrimidinic Endonuclease Ape1L. PLoS ONE 9(3): e92963. doi:10.1371/journal.pone.0092963
