# Supplementary material for: Cloning and Characterization of a Wheat Homologue of Apurinic/Apyrimidinic Endonuclease Ape1L
Source: PLoS One. 2014 Mar 25;9(3):e92963. doi: 10.1371/journal.pone.0092963 (PMC3965494; doi:10.1371/journal.pone.0092963)
Supplement: File S1 — Figures S1–S6. Figure S1. SDS-PAGE analysis of the purified recombinant TaApe1 protein. Lane 1, protein size markers; lane 2, human APE1, 2 μg; lane 3, TaApe1, 1 μg; lane 4, TaApe1, 5 μg. Figure S2. Protein sequence alignment of human APE1, Arabidopsis thaliana AP endonucleases AtApe1L, AtArp and AtApe2. The deduced amino acid sequences were aligned using ClustalX 2.1. Asterisks (*), colons (:), and periods (.) indicate identical, conservative, and semi-conservative aligned residues, respectively. Figure S3. Divalent cation dependence of wheat TaApe1-catalyzed activities on the oligonucleotide duplex THF•T. 10 nM 5′-32P-labelled 30-mer DNA duplex containing a single THF residue was incubated for 10 min at 23°C with 5 or 10 nM TaApe1 under standard reaction conditions but in the presence of different divalent cations. (A) Effects of MgCl2, CaCl2 and CoCl2 on the enzyme activities (B) Effects of ZnCl2, NiCl2 and FeCl2 on the enzyme activities. Lanes 1, 3, 7, 11, control 30-mer duplex THF•T incubated in reaction buffer without enzyme; lane 2, as lane 1 but incubated with 1 nM APE1 for 5 min at 37°C; lanes 4–6, 8–10 and 12–14, 30-mer duplex THF•T incubated with TaApe1L in the presence of the indicated metal chloride. For details, see Materials and Methods. Figure S4. Effects of the absence of divalent cations and/or presence of 1 mM EDTA in the reaction buffer on the TaApe1L AP endonuclease activity. 10 nM 5′-32P-labelled 30-mer DNA duplex containing a single THF residue was incubated with 5 nM TaApe1L at 23°C. Lane 1, control non-treated THF•T duplex; lane 2, as lane 1 but incubated for 5 min at 37°C with 1 nM APE1; lane 3, as lane 2 but incubated with 5 nM TaApe1L and 1 mM MnCl2 for 5 min at 23°C; lane 4, as lane 3 but incubated with 10 nM TaApe1L: lane 5, as lane 3 but incubated with 0 mM MnCl2; lane 6, as lane 5 but incubated with 10 nM TaApe1L; lane 7, as lane 3 but incubated with 1 mM EDTA; lane 8, as lane 7 but incubated with 10 nM TaApe1; lane 9, as lane 3 but i [file pone.0092963.s001.pdf]

Cloning and Characterization of a Wheat Homologue of Apurinic/Apyrimidinic Endonuclease Ape1L

Botagoz JOLDYBAYEVA[a,g], *Paulina PROROK*[b,c,g], *Inga R. GRIN*[b,d,e], *Dmitry O. ZHARKOV*[d,e], *Alexander A. ISHCHENKO*[b], *Barbara TUDEK*[c,f], *Amangeldy K. BISSENBAEV*[a,]* *and Murat SAPARBAEV*[b,]*

[a]*Department of Molecular Biology and Genetics, Faculty of Biology, al-Farabi Kazakh National University, 530038, Almaty, Kazakhstan.*
[b]*Groupe «Réparation de l'ADN», CNRS UMR8200, Université Paris-Sud, Institut Gustave Roussy, F-94805 Villejuif Cedex, France.*
[c]*Institute of Biochemistry and Biophysics, Polish Academy of Sciences, 02-106 Warsaw, Poland.*
[d]*SB RAS Institute of Chemical Biology and Fundamental Medicine, Novosibirsk 630090, Russia*
[e]*Novosibirsk State University, Novosibirsk 630090, Russia*
[f]*Institute of Genetics and Biotechnology, University of Warsaw, 02-106 Warsaw, Poland.*

**Corresponding authors. M.S and A.K.B.: phone: 33 1 42115404; Email: smurat@igr.fr and phone: +77273773437. E-mail address: Amangeldy.Bisenbaev@kaznu.kz*

Running title: *Characterization of the wheat AP endonuclease*

Keywords: DNA repair, oxidative DNA damage; apurinic/apyrimidinic sites, base excision repair, AP endonuclease, 3'-repair diesterase.

Author Contributions: [g]The authors wish it to be known that, in their opinion, the first two authors should be regarded as joint First Authors.

Supporting Information Legend
Supporting Information Figure Legends

Supporting Information Figure S1. SDS-PAGE analysis of the purified recombinant TaApe1 protein. Lane 1, protein size markers; lane 2, human APE1, 2 μg; lane 3, TaApe1, 1 μg; lane 4, TaApe1, 5 μg.

Supporting Information Figure S2. Protein sequence alignment of human APE1, *Arabidopsis thaliana* AP endonucleases AtApe1L, AtArp and AtApe2. The deduced amino acid sequences were aligned using ClustalX 2.1. Asterisks (*), colons (:), and periods (.) indicate identical, conservative, and semi-conservative aligned residues, respectively.

Supporting Information Figure S3. Divalent cation dependence of wheat TaApe1-catalyzed activities on the oligonucleotide duplex THF•T. 10 nM 5'-^{32}P-labelled 30-mer DNA duplex containing a single THF residue was incubated for 10 min at 23°C with 5 or 10 nM TaApe1 under standard reaction conditions but in the presence of different divalent cations. (**A**) Effects of $MgCl_2$, $CaCl_2$ and $CoCl_2$ on the enzyme activities (**B**) Effects of $ZnCl_2$, $NiCl_2$ and $FeCl_2$ on the enzyme activities. Lanes 1, 3, 7, 11, control 30-mer duplex THF•T incubated in reaction buffer without enzyme; lane 2, as lane 1 but incubated with 1 nM APE1 for 5 min at 37°C; lanes 4–6, 8–10 and 12–14, 30-mer duplex THF•T incubated with TaApe1L in the presence of the indicated metal chloride. For details, see Materials and Methods.

Supporting Information Figure S4. Effects of the absence of divalent cations and/or presence of 1 mM EDTA in the reaction buffer on the TaApe1L AP endonuclease activity. 10 nM 5'-^{32}P-labelled 30-mer DNA duplex containing a single THF residue was incubated with 5 nM TaApe1L at 23°C. Lane 1, control non-treated THF•T duplex; lane 2, as lane 1 but incubated for 5 min at 37°C with 1 nM APE1; lane 3, as lane 2 but incubated with 5 nM TaApe1L and 1 mM $MnCl_2$ for 5 min at 23°C; lane 4, as lane 3 but incubated with 10 nM TaApe1L: lane 5, as lane 3 but incubated with 0 mM $MnCl_2$; lane 6, as lane 5 but incubated with 10 nM TaApe1L; lane 7, as lane 3 but incubated with 1 mM EDTA; lane 8, as lane 7 but incubated with 10 nM TaApe1; lane 9, as lane 3 but incubated with 5 mM EDTA; lane 10, as lane 9 but incubated with 10 nM TaApe1. The arrows mark the position of the 30-mer DNA substrate and 10-mer cleavage product. For details, see Materials and Methods.

Supporting Information Figure S5. Preparation of the DNA substrates containing a nick flanked with 3'-PA or 3'-OH termini or a 1-nt gap flanked with 3'-P to measure 3'-repair phoshodiesterase activities of TaApe1L. (**A**) Schematic representation of the structures of 3' and 5' DNA termini at a DNA nick or gap. (**B**) Separation of 5'-^{32}P-labelled oligonucleotide fragments by denaturing PAGE. 10 nM 5'-^{32}P-labelled 34-mer DNA duplex containing a single U•G base pair was incubated for 5 min at 37°C with 10 nM hUNG and then with either 20 nM Fpg or 20 nM Nth or 20 nM Nfo for 15 min at 37°C. Lane 1, control non-treated 34-mer duplex U•G; lane 2, as lane 1 but incubated with 10 nM hUNG and 20 nM Fpg; lane 3, as lane 1 but incubated with 10 nM hUNG and 20 nM Nth; lane 4, as lane 1 but incubated with 10 nM hUNG and 20 nM Nfo. The arrows mark the position of the full-length 34-mer substrate and 19-mer cleavage fragments containing by 3'-PA, 3'-OH and 3'-P. For details, see Materials and Methods.

Supporting Information Figure S6. Activities of human APE1 and TaApe1L on αdA•T and DHU•G oligonucleotide duplexes. 10 nM 5'-^{32}P-labelled 30-mer DNA duplex containing a single modified residue was incubated with 5 nM TaApe1L at 23°C. Lane 1, control non-treated THF•T duplex; lane 2, as lane 1 but treated with 1 nM APE1 for 5 min at 37°C; lane 3, as lane 1 but treated with 5 nM TaApe1L for 5 min; lane 4, as lane 3 but treated for 10 min; lane 5, as lane 3 but treated for 15 min; lane 6, as lane 3 but treated for 30 min; lane 7, control non-treated αdA•T duplex; lane 8, as lane 7 but treated with 5 nM APE1 for 5 min at 37 °C; lane 9, as lane 7 but treated with 5 nM TaApe1L for 5 min; lane 10, as lane 9 but treated for 10 min, lane 11, as lane 9 but treated for 15 min, lane 12, as lane 9 but treated for 30 min; lane 13, control non-treated DHU•G duplex; lane 14, as lane 13 but treated with 5 nM APE1 for 5 min at 37 °C; lane 15, as lane 13 but treated with 5 nM TaApe1L for treated for 5 min; lane 16, as lane 15 but treated for 10 min, lane 17, as lane 15 but treated for 15 min, lane 18, as lane 15 but treated for 30 min. The arrows mark the position of the full-length 30-mer substrate and 10-mer AP-endonuclease and NIR cleavage products. For details, see Materials and Methods.

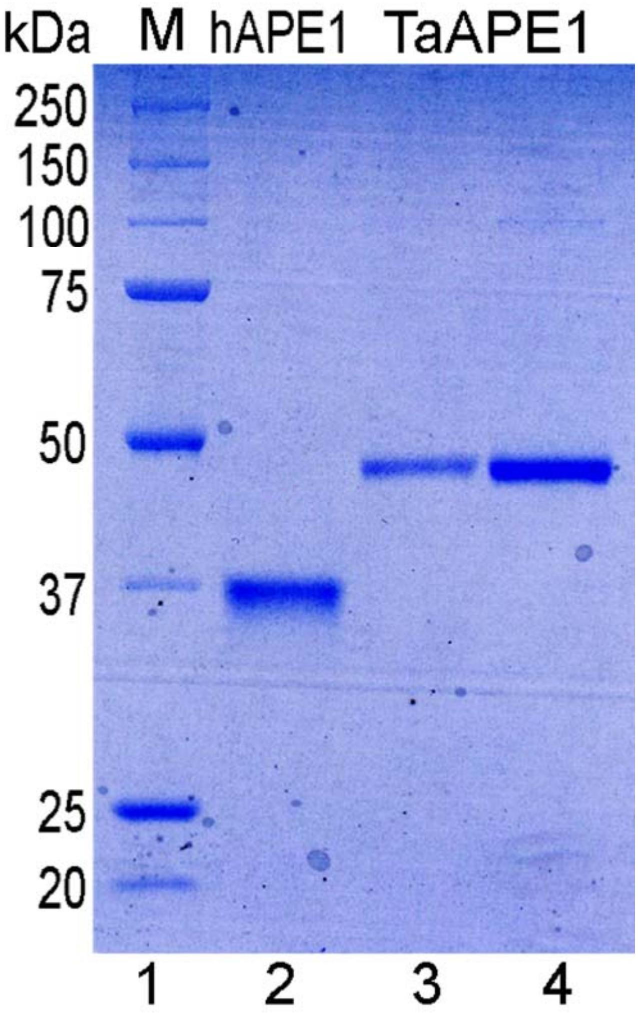

Supporting Information Figure S1. SDS-PAGE analysis of the purified recombinant TaApe1 protein. Lane 1, protein size markers; lane 2, human APE1, 2 µg; lane 3, TaApe1, 1 µg; lane 4, TaApe1, 5 µg.

```
AtArp       MNNALQFGLQSSAIYVAKFLVVPLRSLRVGSSFVGVGVGTRSFNKRLMSNATAFSINNSKRKELNIPGAAIDQNCHQMGSDTDRDEMGTLQDDRKEIEAM 100

AtArp       TVQELRSTLRKLGVPVKGRKQELISTLRLHMDSNLPDQKETSSSTRSDSVTIKRKISNREEPTEDECTNSEAYDIEHGEKRVKQSTEKNLKAKVSAKAIA 200
APE1        ----------------------------------------------------------------------------MPKRGKKGAV--AEDGD-ELR---  18

AtApe2      ----------------------------------------------------------------------------MKIVTYNVNGLRQRVSQFD-SLLK  23
AtApe1L     ------MKRFFKPIEKEN---SPAA------------KKPCLS------------PE-KRDGDGDGVEEEKNQNEPSKFMTWNANSFLLRVKNDWSQFSK  66
AtArp       KEQKSLMRTGKQQI-QSKEETSSTISSKLLKTEEIISSPSQSEPWTVLAHKKPQKDWKAYNPKTMRPPPLPEGTKCVKVMTWNVNGLRGLLKFESFSALQ 299
APE1        ------------TEPEAKKSKTAAKK--------------------NDKEAAG-EGPALYEDPPDQKTSPSGKPATLKICSWNVDGLRAWIKKKG---LD  82
                                                                                         *. ::*.:.:   :.       .

AtApe2      LLDSFDADIICFQETKLRRQELT-----------------ADL--AI-------ADGYESFFSCTRTSEKGRTGYSGVATFCRVKSASSSCETALPVTAE  97
AtApe1L     FVSDFDPDVIAIQEVRMPAAGGKGKPKNHEELSDDTKVLREEKQILTRALSSPPFGNYGVWWSLA------DSKYAGTALLVKKCFK--------PRKVY 152
AtArp       LAQRENFDILCLQETKLQVKDVE-----------------EIKKTLI-------DGYDHSFWSCS----VSKLGYSGTAIISR--IK--------PLSVR 361
APE1        WVKEEAPDILCLQETKCSENKLP-----------------AEL-QEL-------PGLSHQYWSAP----SDKEGYSGVGLLSR--QC--------PLKVS 143
              .    *::.:**.:                                       .    ::* .         *:*.. : :            * ..

AtApe2      EGITGLVNSNSRGGKSETSTVAEGLEEYEKEELLMIDQEGRCVITDHGHFVVFNVYGPRAVADDADRIEFK--HRFYGVLERRWECLLRQGRRVFVVGDL 195
AtApe1L     FNLD-----------------------KLA---SKHEPDGRVILAEFETFRLLNTYSPNNGWKDE-ENAFQRRRKWDKRIVEFLN--KTSDKPLIWCGDL 223
AtArp       YGTG-----------------------LSG---SDHDTEGRIVTAEFDSFYLINTYVPNSGDGLK-RLSYRI-EEWDRTLSNHI-KELEKSKPVVLTGDL 432
APE1        YGIG-----------------------DE-----EHDQEGRVIVAEFDSFVLVTAYVPNAGRGLV-RLEYR--QRWDEAFRKFL-KGLASRKPLVLCGDL 211
             .                                  : :** : ::.  * :...* *.       .  ::  ..:   : .       . : :.  ***

AtApe2      NIAPFAMDRCE--A------------------GPDFEKNEFRKWFRSLLVERGGSFSDVFRSKHPERK--DAFTCWSSSSGAEQFNYGSRIDHILVAGSC 273
AtApe1L     NVSHEEIDVSHPEFFATAKLNGYVPPNKEDCGQPGFTP-SERGRFGATIKE--GRLVDAYRYLHKEQEMESGFSWSG-NPIGKYRGKRMRIDYFLVSEQL 319
AtArp       NCAHEEIDIFNPAGNK-R--------------SAGFTE-EERQSFGANFLD--KGFVDTFRKQHPGV---VGYTYWGYRHGGRKTNKGWRLDYFLVSQSI 511
APE1        NVAHEEIDLRNPKGNK-K--------------NAGFTP-QERQGFGELLQA--VPLADSFRHLYPNTP--YAYTFWTYMMNARSKNVGWRLDYFLLSHSL 291
            * :   :*  .                      ..*   . *  *   :      : * :*  :       .::       ..  .   *:*::*:: .

AtApe2      LHQDEDKQGHSFLACHVKECDILTEYKRFKNENMPTRWKGGLVTKFKGSDHVPVFISFDDLPDIPEHSTP-PLASRYLPMIYGFQQTLVSVFKKRRANEE 372
AtApe1L     KDRIVSCKM---------------------------HGRGIELEGFHGSDHCPVTLELSKPSSEMEQNQVSN                             364
AtArp       AANVHDS-------------------------------Y--ILPDINGSDHCPIGLILKL                                         538
APE1        LPALCDS-------------------------------K--IRSKALGSDHCPITLYLAL                                         318
                 .                                         **** *: : :

AtApe2      AKAIEVSCSSSTQSNTSSICGDISTGPLRNCGSMGISLEKSCSFENKSTSGVTEAETVAATGSIDNLSDGIRASSVRALNISRDGDRKKARKIQSSQLSL 472

AtApe2      KSFFTTNSKVNNVEDSSSSYVSSSPSSQVESITEPNVSGKEDSEPTTSTQEQDQTGSSAKQKNDAALMEWQRIQNLMQNSIPLCKGHKEACVARVVKKPG 572

AtApe2      PTFGRRFYVCSRAEKQTVVISNGLHQNSETSKRVMRSK                                                               610
```

Supporting Information Figure S2. Protein sequence alignment of human APE1, *Arabidopsis thaliana* AP endonucleases AtApe1L, AtArp and AtApe2. The deduced amino acid sequences were aligned using ClustalX 2.1. Asterisks (*), colons (:), and periods (.) indicate identical, conservative, and semi-conservative aligned residues, respectively.

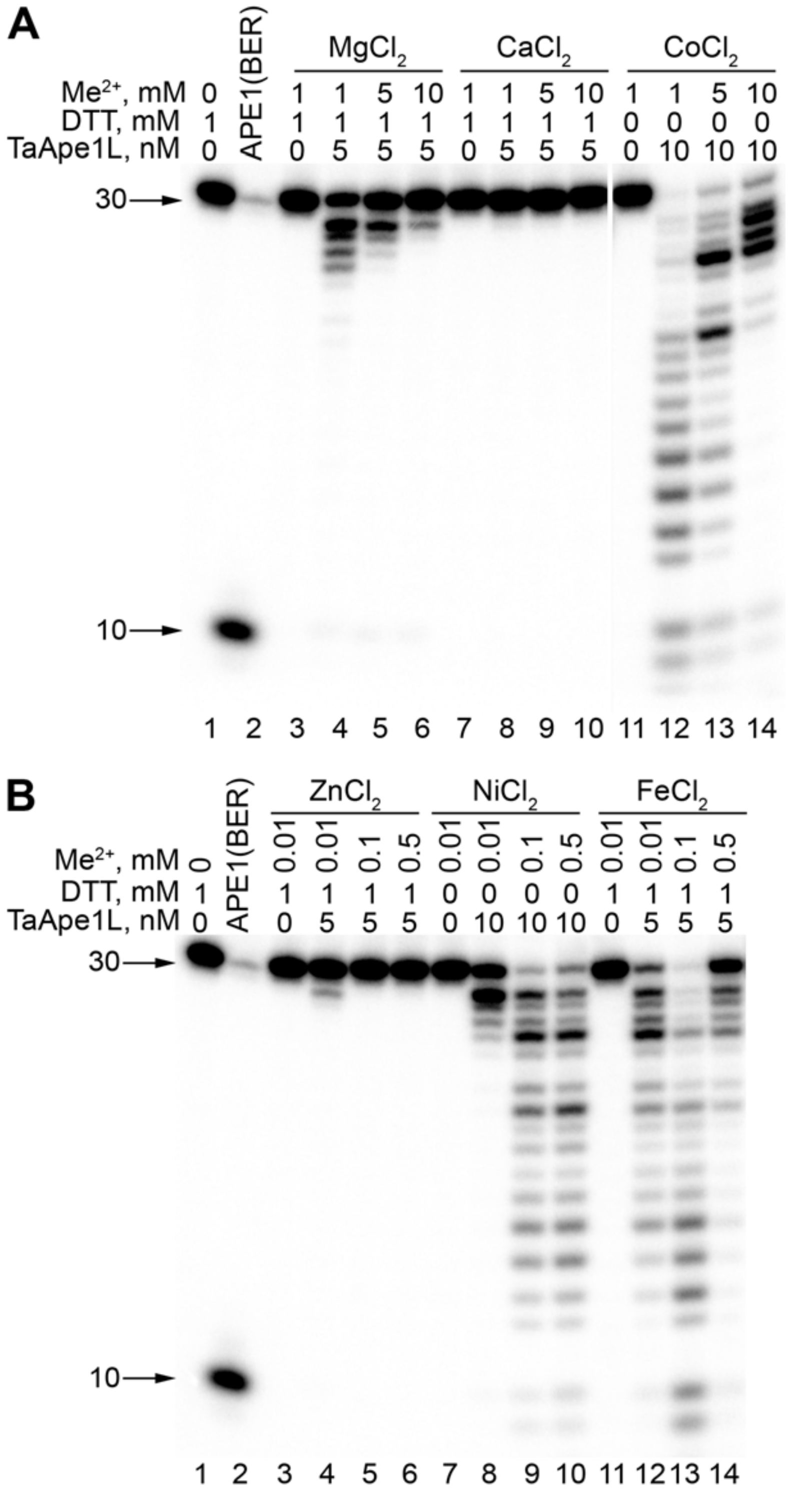

Supporting Information Figure S3. Divalent cation dependence of wheat TaApe1-catalyzed activities on the oligonucleotide duplex THF•T. 10 nM 5'-^{32}P-labelled 30-mer DNA duplex containing a single THF residue was incubated for 10 min at 23°C with 5 or 10 nM TaApe1 under standard reaction conditions but in the presence of different divalent cations. (**A**) Effects of $MgCl_2$, $CaCl_2$ and $CoCl_2$ on the enzyme activities (**B**) Effects of $ZnCl_2$, $NiCl_2$ and $FeCl_2$ on the enzyme activities. Lanes 1, 3, 7, 11, control 30-mer duplex THF•T incubated in reaction buffer without enzyme; lane 2, as lane 1 but incubated with 1 nM APE1 for 5 min at 37°C; lanes 4–6, 8–10 and 12–14, 30-mer duplex THF•T incubated with TaApe1L in the presence of the indicated metal chloride. For details, see Materials and Methods.

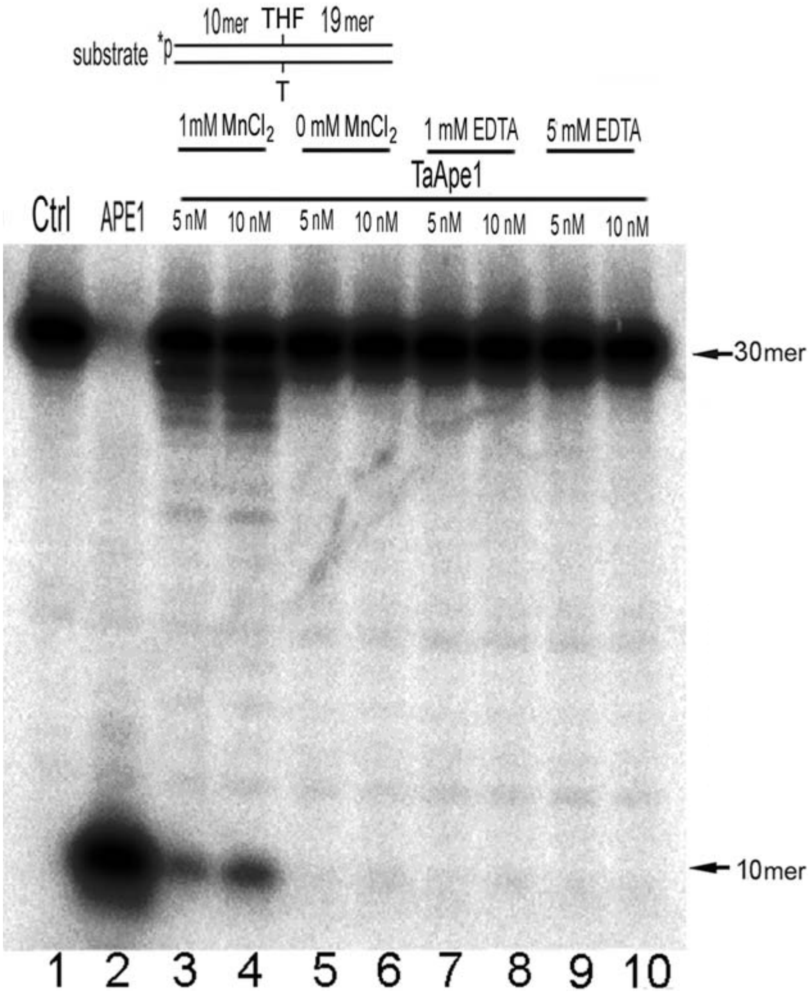

Supporting Information Figure S4. Effects of the absence of divalent cations and/or presence of 1 mM EDTA in the reaction buffer on the TaApe1L AP endonuclease activity. 10 nM 5'-^{32}P-labelled 30-mer DNA duplex containing a single THF residue was incubated with 5 nM TaApe1L at 23°C. Lane 1, control non-treated THF•T duplex; lane 2, as lane 1 but incubated for 5 min at 37°C with 1 nM APE1; lane 3, as lane 2 but incubated with 5 nM TaApe1L and 1 mM $MnCl_2$ for 5 min at 23°C; lane 4, as lane 3 but incubated with 10 nM TaApe1L: lane 5, as lane 3 but incubated with 0 mM $MnCl_2$; lane 6, as lane 5 but incubated with 10 nM TaApe1L; lane 7, as lane 3 but incubated with 1 mM EDTA; lane 8, as lane 7 but incubated with 10 nM TaApe1; lane 9, as lane 3 but incubated with 5 mM EDTA; lane 10, as lane 9 but incubated with 10 nM TaApe1. The arrows mark the position of the 30-mer DNA substrate and 10-mer cleavage product. For details, see Materials and Methods.

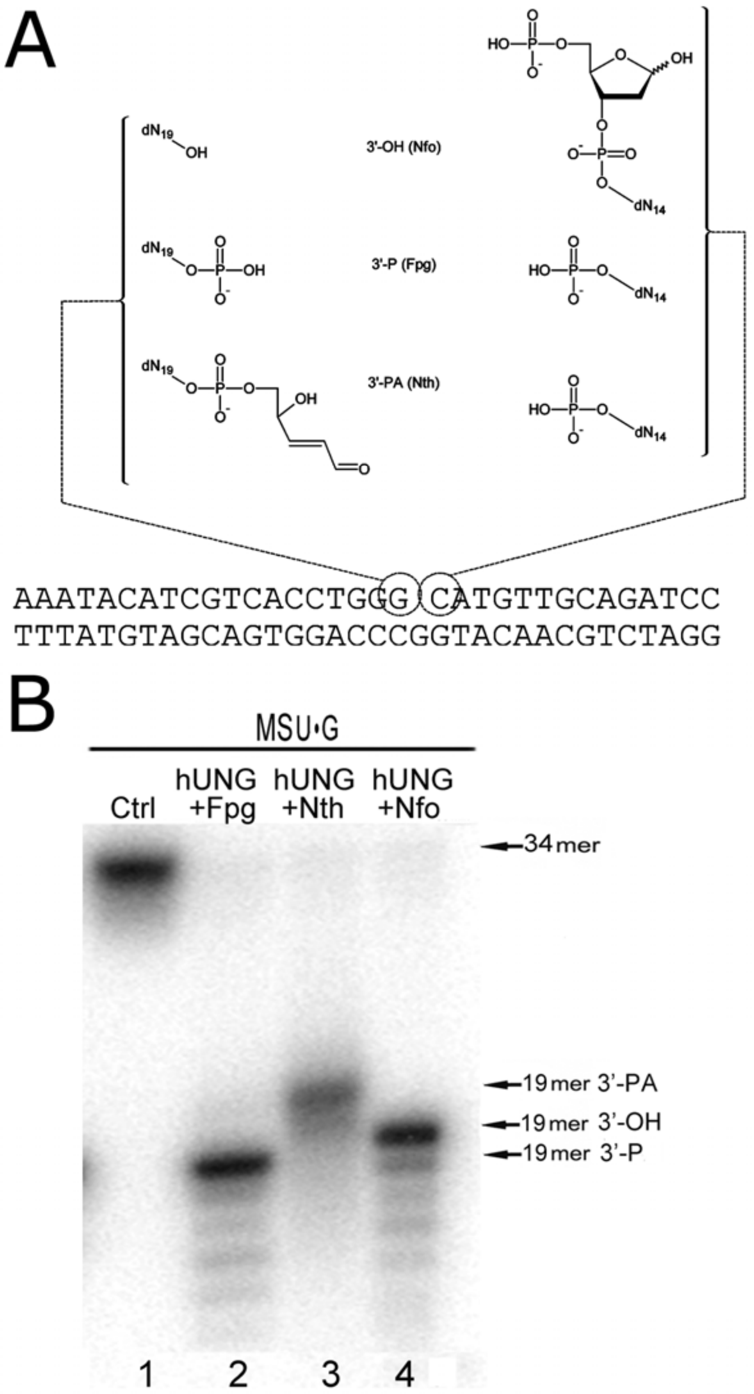

Supporting Information Figure S5. Preparation of the DNA substrates containing a nick flanked with 3'-PA or 3'-OH termini or a 1-nt gap flanked with 3'-P to measure 3'-repair phoshodiesterase activities of TaApe1L. (**A**) Schematic representation of the structures of 3' and 5' DNA termini at a DNA nick or gap. (**B**) Separation of 5'-^{32}P-labelled oligonucleotide fragments by denaturing PAGE. 10 nM 5'-^{32}P-labelled 34-mer DNA duplex containing a single U•G base pair was incubated for 5 min at 37°C with 10 nM hUNG and then with either 20 nM Fpg or 20 nM Nth or 20 nM Nfo for 15 min at 37°C. Lane 1, control non-treated 34-mer duplex U•G; lane 2, as lane 1 but incubated with 10 nM hUNG and 20 nM Fpg; lane 3, as lane 1 but incubated with 10 nM hUNG and 20 nM Nth; lane 4, as lane 1 but incubated with 10 nM hUNG and 20 nM Nfo. The arrows mark the position of the full-length 34-mer substrate and 19-mer cleavage fragments containing by 3'-PA, 3'-OH and 3'-P. For details, see Materials and Methods.

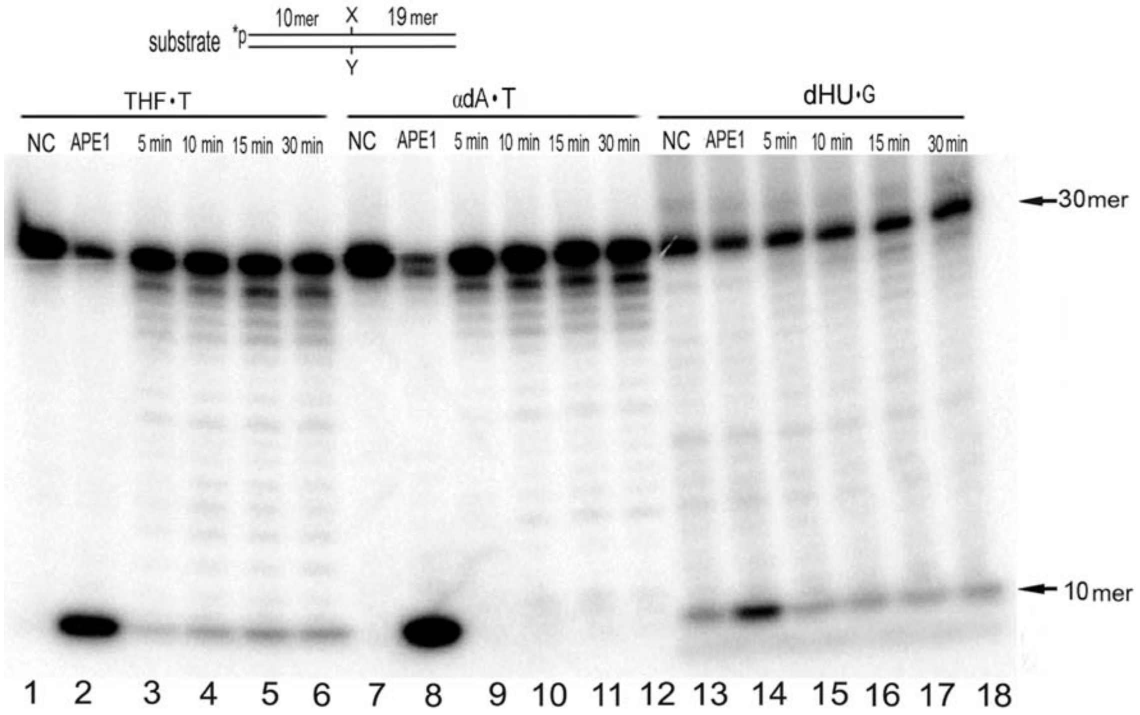

Supporting Information Figure S6. Activities of human APE1 and TaApe1L on αdA•T and DHU•G oligonucleotide duplexes. 10 nM 5'-^{32}P-labelled 30-mer DNA duplex containing a single modified residue was incubated with 5 nM TaApe1L at 23°C. Lane 1, control non-treated THF•T duplex; lane 2, as lane 1 but treated with 1 nM APE1 for 5 min at 37°C; lane 3, as lane 1 but treated with 5 nM TaApe1L for 5 min; lane 4, as lane 3 but treated for 10 min; lane 5, as lane 3 but treated for 15 min; lane 6, as lane 3 but treated for 30 min; lane 7, control non-treated αdA•T duplex; lane 8, as lane 7 but treated with 5 nM APE1 for 5 min at 37 °C; lane 9, as lane 7 but treated with 5 nM TaApe1L for 5 min; lane 10, as lane 9 but treated for 10 min, lane 11, as lane 9 but treated for 15 min, lane 12, as lane 9 but treated for 30 min; lane 13, control non-treated DHU•G duplex; lane 14, as lane 13 but treated with 5 nM APE1 for 5 min at 37 °C; lane 15, as lane 13 but treated with 5 nM TaApe1L for treated for 5 min; lane 16, as lane 15 but treated for 10 min, lane 17, as lane 15 but treated for 15 min, lane 18, as lane 15 but treated for 30 min. The arrows mark the position of the full-length 30-mer substrate and 10-mer AP-endonuclease and NIR cleavage products. For details, see Materials and Methods.
